# Genome-wide analyses of individual differences in quantitatively assessed reading- and language-related skills in up to 34,000 people

**DOI:** 10.1073/pnas.2202764119

**Published:** 2022-08-23

**Authors:** Else Eising, Nazanin Mirza-Schreiber, Eveline L. de Zeeuw, Carol A. Wang, Dongnhu T. Truong, Andrea G. Allegrini, Chin Yang Shapland, Gu Zhu, Karen G. Wigg, Margot L. Gerritse, Barbara Molz, Gökberk Alagöz, Alessandro Gialluisi, Filippo Abbondanza, Kaili Rimfeld, Marjolein van Donkelaar, Zhijie Liao, Philip R. Jansen, Till F. M. Andlauer, Timothy C. Bates, Manon Bernard, Kirsten Blokland, Milene Bonte, Anders D. Børglum, Thomas Bourgeron, Daniel Brandeis, Fabiola Ceroni, Valéria Csépe, Philip S. Dale, Peter F. de Jong, John C. DeFries, Jean-François Démonet, Ditte Demontis, Yu Feng, Scott D. Gordon, Sharon L. Guger, Marianna E. Hayiou-Thomas, Juan A. Hernández-Cabrera, Jouke-Jan Hottenga, Charles Hulme, Juha Kere, Elizabeth N. Kerr, Tanner Koomar, Karin Landerl, Gabriel T. Leonard, Maureen W. Lovett, Heikki Lyytinen, Nicholas G. Martin, Angela Martinelli, Urs Maurer, Jacob J. Michaelson, Kristina Moll, Anthony P. Monaco, Angela T. Morgan, Markus M. Nöthen, Zdenka Pausova, Craig E. Pennell, Bruce F. Pennington, Kaitlyn M. Price, Veera M. Rajagopal, Franck Ramus, Louis Richer, Nuala H. Simpson, Shelley D. Smith, Margaret J. Snowling, John Stein, Lisa J. Strug, Joel B. Talcott, Henning Tiemeier, Marc P. van der Schroeff, Ellen Verhoef, Kate E. Watkins, Margaret Wilkinson, Margaret J. Wright, Cathy L. Barr, Dorret I. Boomsma, Manuel Carreiras, Marie-Christine J. Franken, Jeffrey R. Gruen, Michelle Luciano, Bertram Müller-Myhsok, Dianne F. Newbury, Richard K. Olson, Silvia Paracchini, Tomáš Paus, Robert Plomin, Sheena Reilly, Gerd Schulte-Körne, J. Bruce Tomblin, Elsje van Bergen, Andrew J. O. Whitehouse, Erik G. Willcutt, Beate St Pourcain, Clyde Francks, Simon E. Fisher

**Affiliations:** ^a^Language and Genetics Department, Max Planck Institute for Psycholinguistics, 6525 XD Nijmegen, the Netherlands;; ^b^Institute of Neurogenomics, Helmholtz Zentrum Munich, 85764 Munich, Germany;; ^c^Department of Biological Psychology, Vrije Universiteit Amsterdam, 1081 BT Amsterdam, the Netherlands;; ^d^School of Medicine and Public Health, The University of Newcastle, Newcastle, NSW 2308, Australia;; ^e^Mothers and Babies Research Program, Hunter Medical Research Institute, Newcastle, NSW 2305, Australia;; ^f^Department of Pediatrics and Genetics, Yale Medical School, New Haven, CT 06510;; ^g^Social, Genetic and Developmental Psychiatry Centre, Institute of Psychiatry, Psychology and Neuroscience, King’s College London, London SE5 8AF, United Kingdom;; ^h^MRC Integrative Epidemiology Unit, University of Bristol, Bristol BS8 2BN, United Kingdom;; ^i^Population Health Sciences, University of Bristol, Bristol BS8 2PS, United Kingdom;; ^j^Genetic Epidemiology Laboratory, QIMR Berghofer Medical Research Institute, Brisbane, QLD 4006, Australia;; ^k^Division of Experimental and Translational Neuroscience, Krembil Research Institute, University Health Network, Toronto, ON M5T 0S8, Canada;; ^l^Translational Research in Psychiatry, Max Planck Institute of Psychiatry, 80804 Munich, Germany;; ^m^Department of Epidemiology and Prevention, IRCCS Istituto Neurologico Mediterraneo Neuromed, 86077 Pozzilli, Italy;; ^n^Department of Medicine and Surgery, University of Insubria, 21100 Varese, Italy;; ^o^School of Medicine, University of St Andrews, KY16 9TF, St. Andrews, Scotland;; ^p^Department of Psychology, Royal Holloway, University of London, Egham TW20 0EY, United Kingdom;; ^q^Department of Psychology, University of Toronto, Toronto, ON M5S 3G3,Canada;; ^r^Department of Child and Adolescent Psychiatry/Psychology, Erasmus University Medical Center, 3000 CB Rotterdam, the Netherlands;; ^s^Department of Complex Trait Genetics, Center for Neurogenomics and Cognitive Research, Amsterdam Neuroscience, Vrije Universiteit Amsterdam, Amsterdam, 1081 HV the Netherlands;; ^t^Department of Human Genetics, VU Medical Center, Amsterdam University Medical Center, 1081 BT Amsterdam, the Netherlands;; ^u^Department of Neurology, Klinikum rechts der Isar, School of Medicine, Technical University of Munich, 81675 Munich, Germany;; ^v^Department of Psychology, University of Edinburgh, Edinburgh EH8 9JZ, United Kingdom;; ^w^Department of Physiology and Nutritional Sciences, University of Toronto, Toronto, ON M5S 1A1, Canada;; ^x^Program in Neuroscience and Mental Health, Hospital for Sick Children, Toronto, M5G 1X8 ON, Canada;; ^y^Department of Cognitive Neuroscience and Maastricht Brain Imaging Center, Faculty of Psychology and Neuroscience, Maastricht University, 6229 ER Maastricht, the Netherlands;; ^z^Department of Biomedicine, Aarhus University, 8000 Aarhus, Denmark;; ^aa^The Lundbeck Foundation Initiative for Integrative Psychiatric Research, iPSYCH, 8210 Aarhus, Denmark;; ^bb^Center for Genomics and Personalized Medicine (CGPM), 8000 Aarhus, Denmark;; ^cc^Human Genetics and Cognitive Functions, Institut Pasteur, UMR3571 Centre national de la recherche scientifique (CNRS), Université Paris Cité, Paris, 75015, France;; ^dd^Department of Child and Adolescent Psychiatry and Psychotherapy, Psychiatric Hospital, University of Zurich, 8032 Zurich, Switzerland;; ^ee^Zurich Center for Integrative Human Physiology, University of Zurich and ETH Zurich, 8057 Zurich, Switzerland;; ^ff^Neuroscience Center Zurich, University of Zurich and ETH Zurich, 8057 Zurich, Switzerland;; ^gg^Department of Child and Adolescent Psychiatry and Psychotherapy, Central Institute of Mental Health, Medical Faculty Mannheim, Heidelberg University, 68159 Mannheim, Germany;; ^hh^Department of Pharmacy and Biotechnology, University of Bologna, 40126 Bologna, Italy;; ^ii^Faculty of Health and Life Sciences, Oxford Brookes University, Oxford OX3 0BP, United Kingdom;; ^jj^Brain Imaging Centre, Research Centre for Natural Sciences, Budapest, 1117 Hungary;; ^kk^Multilingualism Doctoral School, Faculty of Modern Philology and Social Sciences, University of Pannonia, Veszprém, 8200 Hungary;; ^ll^Department of Speech & Hearing Sciences, University of New Mexico, Albuquerque, NM 87131;; ^mm^Department of Child Development and Education, University of Amsterdam, 1012 WX Amsterdam, the Netherlands;; ^nn^Institute for Behavioral Genetics, University of Colorado, Boulder, CO 80309-0447;; ^oo^Department of Psychology and Neuroscience, University of Colorado, Boulder, CO 80309-0447;; ^pp^Leenaards Memory Centre, Department of Clinical Neurosciences, Lausanne University Hospital (CHUV), University of Lausanne, CH-1011 Lausanne, Switzerland;; ^qq^Department of Psychology, Hospital for Sick Children, Toronto, ON M5G 1X8, Canada;; ^rr^Department of Psychology, University of York, York YO10 5DD, United Kingdom;; ^ss^Departamento de Psicología, Clínica Psicobiología y Metodología, 38200, La Laguna, Santa Cruz de Tenerife, Spain;; ^tt^Department of Education, University of Oxford, Oxford, Oxfordshire OX2 6PY, United Kingdom;; ^uu^Department of Biosciences and Nutrition, Karolinska Institutet, 171 77 Stockholm, Sweden;; ^vv^Stem Cells and Metabolism Research Program, University of Helsinki and Folkhälsan Research Center, 00014 Helsinki, Finland;; ^ww^Department of Neurology, Hospital for Sick Children, Toronto, ON M5G 1X8, Canada;; ^xx^Department of Paediatrics, University of Toronto, Toronto, ON M5G 1X8, Canada;; ^yy^Department of Psychiatry, University of Iowa, Iowa City, IA 52242;; ^zz^Institute of Psychology, University of Graz, 8010 Graz, Austria;; ^aaa^BioTechMed-Graz, 8010 Graz, Austria;; ^bbb^Cognitive Neuroscience Neurology and Neurosurgery, McGill University, Montreal, QC H3A 1G1, Canada;; ^ccc^Department of Psychology, University of Jyväskylä, 40014 Jyväskylä, Finland;; ^ddd^Department of Psychology, The Chinese University of Hong Kong, Hong Kong, China;; ^eee^Department of Child and Adolescent Psychiatry, Psychosomatics, and Psychotherapy, Ludwig-Maximilians-University Hospital Munich, Munich, 80336 Germany;; ^fff^Office of the President, Tufts University, Medford, MA 02155;; ^ggg^Speech and Language, Murdoch Children's Research Institute, Melbourne, VIC 3052, Australia;; ^hhh^Department of Audiology and Speech Pathology, University of Melbourne, Melbourne, VIC 3052, Australia;; ^iii^Speech Pathology Department, Royal Children's Hospital, Melbourne, VIC 3052, Australia;; ^jjj^Institute of Human Genetics, University Hospital of Bonn, 53127 Bonn, Germany;; ^kkk^Hospital for Sick Children, Toronto, ON M5G 1X8, Canada;; ^lll^Maternity and Gynaecology, John Hunter Hospital, Newcastle, NSW 2305, Australia;; ^mmm^Department of Psychology, University of Denver, Denver, CO 80210;; ^nnn^Department of Physiology, University of Toronto, Toronto, ON M5S 1A8, Canada;; ^ooo^Laboratoire de Sciences Cognitives et Psycholinguistique, Ecole Normale Supérieure, Paris Sciences & Lettres University, École des Hautes Études en Sciences Sociales (EHESS), Centre National de la Recherche Scientifique (CNRS), Paris, 75005 France;; ^ppp^Department of Health Sciences, Université du Québec à Chicoutimi, Chicoutimi, QC G7H 2B1, Canada;; ^qqq^Department of Experimental Psychology, University of Oxford, Oxford OX2 6GG, United Kingdom;; ^rrr^Department of Neurological Sciences, College of Medicine, University of Nebraska Medical Center, Omaha, NE 68198;; ^sss^St. John’s College, University of Oxford, Oxford OX1 3JP, United Kingdom;; ^ttt^Department of Physiology, Anatomy and Genetics, Oxford University, Oxford OX1 3PT, United Kingdom;; ^uuu^Department of Statistical Sciences and Computer Science and Division of Biostatistics, University of Toronto, Toronto, ON M5S 3G3, Canada;; ^vvv^Program in Genetics and Genome Biology and the Centre for Applied Genomics, Hospital for Sick Children, Toronto, ON M5G 1X8, Canada;; ^www^Institute for Health and Neurodevelopment, Aston University, Birmingham B4 7ET, United Kingdom;; ^xxx^T. H. Chan School of Public Health, Harvard, Boston, MA 02115;; ^yyy^Department of Otolaryngology, Head and Neck Surgery, Erasmus University Medical Center, 3015 GD Rotterdam, the Netherlands;; ^zzz^Generation R Study Group, Erasmus University Medical Center, 3015 GD Rotterdam, the Netherlands;; ^aaaa^Queensland Brain Institute, University of Queensland, Brisbane, QLD 4072, Australia;; ^bbbb^Netherlands Twin Register, 1081 BT Amsterdam, the Netherlands;; ^cccc^Amsterdam Reproduction and Development Research Institute, Amsterdam University Medical Center, 1105 AZ Amsterdam, the Netherlands;; ^dddd^Basque Center on Cognition, Brain and Language, Donostia-San Sebastian, 20009 Gipuzkoa, Spain;; ^eeee^Ikerbasque, Basque Foundation for Science, 48009 Bilbao, Vizcaya, Spain;; ^ffff^Lengua Vasca y Comunicación, University of the Basque Country, 48940 Bilbao, Vizcaya, Spain;; ^gggg^Department of Health Science, University of Liverpool, Liverpool L69 7ZX, United Kingdom;; ^hhhh^Department of Psychiatry and Neuroscience and Centre Hospitalier Universitaire Sainte Justine, University of Montreal, Montreal, QC H3T 1J4, Canada;; ^iiii^Menzies Health Institute Queensland, Griffith University, Gold Coast, QLD 4222, Australia;; ^jjjj^Communication Sciences and Disorders, University of Iowa, Iowa City, IA 52242;; ^kkkk^Research Institute LEARN!, Vrije Universiteit Amsterdam, 1081 BT Amsterdam, the Netherlands;; ^llll^Telethon Kids Institute, The University of Western Australia, Perth, WA 6009, Australia;; ^mmmm^Donders Institute for Brain, Cognition and Behaviour, Radboud University, 6525 EN Nijmegen, the Netherlands;; ^nnnn^Department of Human Genetics, Radboud University Medical Center, 6525 GA Nijmegen, the Netherlands

**Keywords:** reading, language, genome-wide association study, meta-analysis

## Abstract

The use of spoken and written language is a fundamental human capacity. Individual differences in reading- and language-related skills are influenced by genetic variation, with twin-based heritability estimates of 30 to 80% depending on the trait. The genetic architecture is complex, heterogeneous, and multifactorial, but investigations of contributions of single-nucleotide polymorphisms (SNPs) were thus far underpowered. We present a multicohort genome-wide association study (GWAS) of five traits assessed individually using psychometric measures (word reading, nonword reading, spelling, phoneme awareness, and nonword repetition) in samples of 13,633 to 33,959 participants aged 5 to 26 y. We identified genome-wide significant association with word reading (rs11208009, *P* = 1.098 × 10^−8^) at a locus that has not been associated with intelligence or educational attainment. All five reading-/language-related traits showed robust SNP heritability, accounting for 13 to 26% of trait variability. Genomic structural equation modeling revealed a shared genetic factor explaining most of the variation in word/nonword reading, spelling, and phoneme awareness, which only partially overlapped with genetic variation contributing to nonword repetition, intelligence, and educational attainment. A multivariate GWAS of word/nonword reading, spelling, and phoneme awareness maximized power for follow-up investigation. Genetic correlation analysis with neuroimaging traits identified an association with the surface area of the banks of the left superior temporal sulcus, a brain region linked to the processing of spoken and written language. Heritability was enriched for genomic elements regulating gene expression in the fetal brain and in chromosomal regions that are depleted of Neanderthal variants. Together, these results provide avenues for deciphering the biological underpinnings of uniquely human traits.

## Significance

Our unique capacities for spoken and written language are fundamental features of what makes us human, yet the biological bases remain largely mysterious. We present a large-scale well-powered genome-wide association study meta-analysis of individual differences in reading- and language-related skills (word reading, nonword reading, spelling, phoneme awareness, and nonword repetition) in tens of thousands of participants. The findings prompt a major reevaluation of prior literature claiming candidate gene associations in much smaller samples. Moreover, we use the novel genetic data as windows into multiple aspects of the biology of these important abilities, revealing molecular links to individual differences in neuroanatomy of language-related brain areas and enriched heritability in archaic deserts of the human genome as well as in fetal brain enhancer regions.

The processing and production of complex spoken and written language are capacities that appear to be distinct to our species (1). Such skills have become fundamental for day-to-day life in modern society. Decades of family and twin studies have revealed substantial genetic components contributing to individual variation in reading- and language-related traits as well as to susceptibility to associated disorders (2). A recent meta-analysis integrated available data on these skills from 49 twin studies, with a total sample size of 38,000 children and adolescents aged 4 to 18 y. The meta-analysis yielded heritability estimates of 66% for word reading (meta-analysis of 48 studies), 80% for spelling (15 studies), and 52% for phoneme awareness (the ability to identify and manipulate individual sounds of spoken words; 13 studies) and suggested greater genetic influences on reading-related abilities than language-related measures (twin heritability of 34%; meta-analysis of 10 studies with measures on receptive and expressive vocabulary, oral language, and naming abilities) (3).

Linkage mapping and targeted candidate gene studies have reported associations of SNPs and/or genetic loci with reading- and language-related traits as well as with disorders such as dyslexia and developmental language disorder (DLD), which encompasses the older definition of specific language impairment (SLI) (4). However, replication efforts have been met with limited success (4). Moreover, for the language sciences, in contrast to other areas of human genetics, there have so far been few genome-wide association studies (GWASs), in which SNPs at millions of points across the genome are systematically screened for association with the trait of interest in large datasets comprising thousands of individuals (2). GWAS efforts are beginning to identify SNPs that show genome-wide significant associations with reading- and language-related traits: rs7642482 near *ROBO2* associated with expressive vocabulary in infancy (5); rs17663182 within *MIR924HG* with rapid automatized naming of letters (6); and rs1555839 near *RPL7P34* with rapid automatized naming and rapid alternating stimulus, deficits of which are often implicated in dyslexia (7). Nonetheless, insights into the genomic underpinnings of these types of skills from GWAS approaches have thus far been limited, which may reflect low power due to the relatively small sample sizes of the cohorts, such that the majority of genetic variance remains unexplained. Sample sizes have remained limited because of the labor-intensive assessment methods required for phenotypic characterization of reading- and language-related traits, which are difficult or even impossible to replace with simple questionnaires. Yet, well-powered GWAS efforts that characterize the molecular genetic variation involved in reading- and language-related traits have the potential to provide novel perspectives on the biological bases and origins of human cognitive specializations (8).

Here, we present large-scale GWAS meta-analyses of a set of reading- and language-related traits, measured with psychometric tools. We captured variation across the phenotypic spectrum, extending beyond disorder. Our study focused on traits assessed using continuous measures in multiple cohorts from the international GenLang network (https://www.genlang.org/) together with several public datasets that have data available for the relevant phenotypes matched to genome-wide genotype information. Five quantitative traits were identified for which phenotype data could be aligned across different cohorts to yield sufficiently large sample sizes for GWAS: word reading, nonword reading, spelling, phoneme awareness, and nonword repetition. Univariate GWAS meta-analyses were performed for each of the phenotypes to identify genetic variation influencing these traits and to model genetic overlaps between them. For comparative purposes, a GWAS meta-analysis for performance intelligence quotient (IQ) was also performed in the same dataset. Together with publicly available GWAS summary statistics from prior studies of cognitive performance and educational attainment, these data were used to study genetic relationships between reading- and language-related traits, IQ, and educational attainment. A multivariate approach allowed us to optimize the power of GWAS meta-analysis for functional follow-ups, giving insights into the tissues, cell types, brain regions, and evolutionary signatures involved.

## Results

### Meta-Analyses of Quantitative Reading- and Language-Related Traits in 22 Cohorts.

Our study focused on five quantitative reading- and language-related traits: word reading accuracy, nonword reading accuracy, spelling accuracy, phoneme awareness, and nonword repetition accuracy (Table 1). These traits are thought to tap into a number of underlying processes involved in written and spoken language. For example, nonword reading relies heavily on basic decoding skills [translating graphemes one by one into phonemes (9)], while spelling utilizes lexical and orthographic knowledge [understanding of permissible letter patterns and how they are arranged in specific words (10)]. Phoneme awareness measures the ability to distinguish and manipulate the separate phonemes in spoken words (11). Nonword repetition tasks tap into speech perception, phonological short-term memory, and articulation (12). A total of 22 cohorts aggregated by the GenLang Consortium combined with several publicly available datasets provided data for one or several of these traits (Datasets S1–S3 and *SI Appendix*, Figs. S1 and S2). The cohorts connected in the GenLang network either were originally ascertained through a proband with a language/reading disorder (DLD/SLI or dyslexia) or were sampled from the general population; all cohorts include quantitative phenotypic data gathered via validated psychometric tests as well as genome-wide genotype data from the tested individuals. Some of the samples are birth cohorts, and some involve family or twin designs. The phenotype data were collected across an array of different ages, test instruments, and languages (primarily English but also, Dutch, Spanish, German, French, Finnish, and Hungarian). We reduced heterogeneity of assessment age by excluding individuals over 18 y of age (except for three cohorts) (*SI Appendix*, *Extended Methods*) and where phenotype data were available from the same participant at multiple ages, by choosing the age that matched best with the assessment ages of the largest cohort(s). We limited the heterogeneity introduced by different test instruments by only including those that measured the phenotypes described in our analysis plan (*SI Appendix*, *Supplemental Notes*) and in cases where data from more than one test instrument were available, by selecting the test instrument that was used by the largest number of cohorts.

**Table 1.** Phenotypes and sample sizes of the GWAS meta-analyses

Trait	Phenotype description	Meta-analysis total sample		Meta-analysis European ancestry only	
		No. of cohorts	No. of individuals	No. of cohorts	No. of individuals
Word reading	Number of correct words read aloud from a list in a time-restricted or unrestricted fashion	19	33,959	18	27,180
Nonword* reading	Number of nonwords read aloud correctly from a list in a time-restricted or unrestricted fashion	13	17,984	12	16,746
Spelling	Number of words correctly spelled orally or in writing after being dictated as single words or in a sentence	15	18,514	14	17,278
Phoneme awareness	Number of words correctly altered in phoneme deletion/elision and spoonerism tasks	12	13,633	11	12,411
Nonword* repetition	Number of nonwords or phonemes repeated aloud correctly	10	14,046	10	12,828

^*^A nonword is a group of phonemes that looks or sounds like a word, obeys the phonotactic rules of the language, but has no meaning.

We evaluated whether, despite efforts to minimize this, heterogeneity related to age and/or use of different test instruments remained in our data. To do so, we generated GWAS meta-analysis results for word and nonword reading stratified by age or test instrument (Dataset S4). We tested those data for genetic correlations using linkage disequilibrium score regression (LDSC) (13), determining to what extent the same common genetic variation is accounting for phenotypic variability in the different stratified GWAS datasets (detailed in *SI Appendix,* *Supplemental Notes*). There was limited heterogeneity of GWAS meta-analysis results, as also evident in the Cochran Q statistics (*SI Appendix*, Fig. S3) and LDSC ratios (Dataset S4) for all traits except nonword repetition.

We also assessed effects of sex, observing high genetic correlations for female- and male-only subsets (*SI Appendix* *Supplemental Notes*, Dataset S4). Given the lack of genetic heterogeneity, the remainder of the study involved analyses of the full non-stratified dataset, to ensure largest available sample size and maximal power.

### A Genome-Wide Significant Locus Associated with Word Reading.

We performed univariate GWAS meta-analyses for the reading-/language-related traits in the full GenLang dataset as follows. For each phenotype, associations between SNPs and the quantitative trait were calculated in every cohort separately, then combined into a meta-analysis for that phenotype. This yielded five separate univariate GWAS datasets, one for each trait. For evaluating statistical significance of SNP associations, we determined an appropriate threshold that was adjusted not only for genome-wide screening (*P* = 5 × 10^−8^), but also for multiple testing based on the correlation structure of our five reading-/language-related traits, as estimated using phenotypic Spectral Decomposition (phenoSpD; *Materials and Methods*). The significance threshold for assessing the GWAS meta-analysis results was thus set to *P* = 5 × 10^−8^/2.15 independent traits = 2.33 × 10^−8^. We identified a genome-wide significant locus associated with word reading (rs11208009 C/T on chromosome 1, *P* = 1.10 × 10^−8^, beta = 0.048, SE = 0.008) (*SI Appendix*, Fig. S5). Notably, rs11208009 has not shown association with general cognitive performance or educational attainment, while other SNPs in linkage disequilibrium (LD) with rs11208009 (*r*^2^ > 0.6) have been associated with triglyceride and total cholesterol levels in blood in previous GWAS (Dataset S5). Three genes are located in the vicinity of rs11208009 and SNPs in LD (*r*^2^ > 0.6): *DOCK7*, encoding a guanine nucleotide exchange factor important for neurogenesis (14); *ANGPTL3*, which encodes a growth factor specific for the vascular endothelium that is expressed specifically in the liver (15); and *USP1*, encoding a deubiquitinating enzyme specific for the Fanconi anemia pathway (16). The associated locus harbors an expression quantitative trait locus regulating *DOCK7* and *ATG4C* [another nearby gene that encodes an autophagy regulator (17)] in the cerebellum and *DOCK7*, *ATG4C*, and *USP1* in blood (Dataset S6). Genome-wide significant loci were not identified for the other traits. Dataset S7 lists all results with *P* < 1 × 10^−6^.

### Traits Related to Written and Spoken Language Are Highly Correlated at the Genetic Level.

**Fig. 1.**
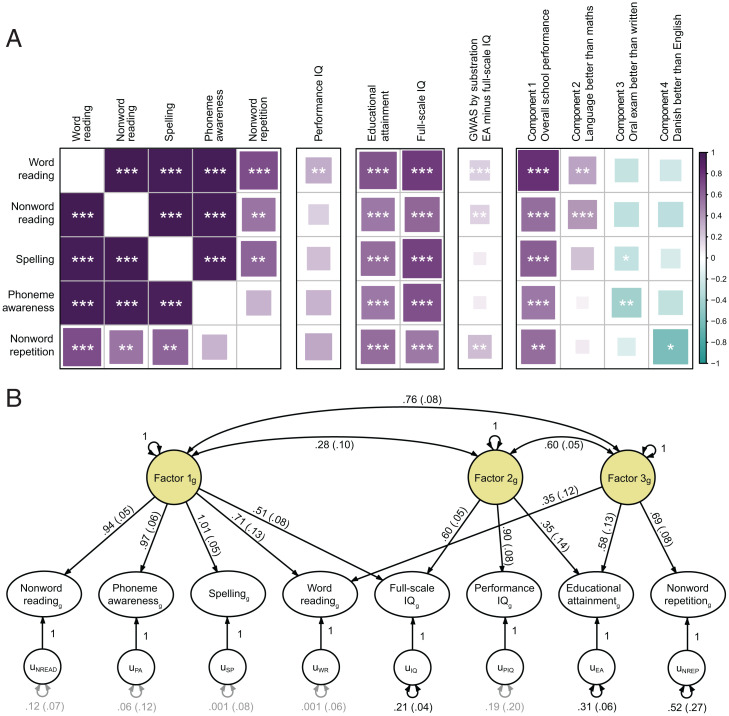
Reading- and language-related traits have a shared genetic architecture that is largely independent of performance IQ. (*A*) Genetic correlations (rg) among the reading- and language-related traits estimated with LDSC. Estimates are capped at one. Full LDSC results are reported in Dataset S4. In addition, genetic correlations are given between the GenLang traits and 1) performance IQ (using GenLang cohorts only); 2) educational attainment (EA; *n* = 766,345) and full-scale IQ (*n* = 257,828) (18); 3) noncognitive abilities involved in EA, resulting from a recent GWAS by subtraction study (*n* = 510,795) (19); and 4) components associated with distinct performance domains identified used a decomposition analysis of Danish school grades (*n* = 30,982) (20). Full results can be found in Dataset S8. *Significant genetic correlation after correction for 18.28 independent comparisons (*P* < 2.74 × 10^−3^); **P < 2.74 × 10^−4^; ***P < 2.74 × 10^−5^. (*B*) Three-factor model fitted to the GenLang summary statistics for word reading, nonword reading, spelling, phoneme awareness, nonword repetition, and performance IQ and to published GWAS summary statistics for full-scale IQ and EA (18) using GenomicSEM (21). Black and gray paths represent factor loadings with *P* < 0.05 and *P* > 0.05, respectively. Standardized factor loadings are shown, with SE in parentheses. The subscript g represents the genetic variables; the u variables represent the residual genetic variance not explained by the models. Unstandardized results and model fit indices are reported in Dataset S9.

The individual effect size of our genome-wide significant hit is small, as is typical for genetically complex traits. We went on to make use of the complete GWAS signal considered in aggregate across the genome to gain insights into the genetic architecture of the reading-/language-related traits as well as relationships with other aspects of human biology. First, for each phenotype, we estimated SNP-based heritability: the proportion of trait variability explained by the SNPs included in the GWAS. All five traits showed significant SNP-based heritability, with LDSC-based estimates ranging from 0.13 for nonword repetition to 0.26 for nonword reading (Dataset S4), indicating that the captured common genetic variation accounts for a substantive proportion of the phenotypic variance. These observations allowed for follow-up analyses that are dependent on significant SNP heritability, including estimates of genetic correlations: a measure that quantifies the overall genetic similarity between two complex traits. Pairwise genetic correlation analyses showed significant overlap among the reading- and language-related traits (Fig. 1*A* and Dataset S4). Genetic correlation estimates were especially high for word reading, nonword reading, spelling, and phoneme awareness, ranging from 0.96 (SE = 0.07) to 1.06 (SE = 0.07).

Prior literature has shown phenotypic correlations of reading- and language-related traits with general cognitive performance and educational attainment. Most cognitive assessments depend on a combination of verbal and nonverbal tests. To enable the investigation of genetic overlaps between nonverbal cognitive performance and reading- and language-related traits while closely matching the sample characteristics of our study, we carried out a GWAS meta-analysis of performance IQ in the GenLang network (*n* = 18,722) (*SI Appendix*, Figs. S1–S3). Only nonverbal subtests of general intelligence tests were used in this analysis (Dataset S1). Summary statistics were also obtained from another three sources: 1) genome-wide studies of full-scale IQ (based on both verbal and nonverbal tasks; *n* = 257,828) and educational attainment (*n* = 766,345) by the Social Science Genetic Association Consortium (18); 2) a GWAS by subtraction study that investigated the noncognitive abilities involved in educational attainment (*n* = 510,795) (19); and 3) a recent GWAS analysis of school grades in the Danish Integrative Psychiatric Research (iPSYCH) cohort (*n* = 30,982) that used a decomposition analysis to identify genetic associations with distinct domains of performance (20).

The five GenLang reading-/language-related traits showed moderate to strong positive genetic correlations with full-scale IQ (range = 0.52 to 0.77), educational attainment (range = 0.54 to 0.68), and school performance (range = 0.54 to 0.81) (Fig. 1*A* and Dataset S8). Interestingly, genetic correlations with full-scale IQ were substantially higher for word reading (95% CI = 0.70 to 0.85) than nonword reading (95% CI = 0.50 to 0.68), likely reflecting the importance of reading skills for verbal tests of cognition. Genetic correlations of reading-/language-related traits with performance IQ (range = 0.20 to 0.35) were much lower than those for full-scale IQ, and the 95% CIs did not overlap. Indeed, only word reading showed a significant genetic correlation with performance IQ. These results indicate that reading-/language-related traits and IQ are at least partly based on distinct genetic factors. Significant trait-specific genetic correlations were observed for components 2 to 4 of the Danish school grade decomposition analysis (20). Component 2, reflecting relatively better school grade performance in language than mathematics (as compared with peers), was positively correlated with both GenLang reading traits. Component 3, reflecting relatively better school grade performance in oral than in written examinations, showed significant negative correlations with phoneme awareness and spelling. Lastly, component 4, reflecting relatively better school grade performance in Danish than in English, showed significant negative correlation with nonword repetition. The noncognitive abilities involved in educational attainment identified in the GWAS by subtraction study (19) showed small but significant positive genetic correlations with word reading, nonword reading, and nonword repetition.

Genomic structural equation modeling (GenomicSEM) (21) is a method that can use GWAS results to fit and compare models describing genetic overlaps between multiple phenotypes to further understand how traits are related. We used this method to model the shared genetic architecture of the five reading- and language-related traits together with performance IQ, full-scale IQ, and educational attainment (Dataset S9). Exploratory factor models with one to four factors were fitted to the data, of which the three-factor model explained the majority of the variance. The three-factor model was followed up using confirmatory factor analysis in GenomicSEM. In the final model (Fig. 1*B*), the first factor explains variation in nonword reading, spelling, phoneme awareness, word reading, and full-scale IQ. For the first three traits, there is no evidence for additional genetic influences, suggesting high genetic similarity. The second factor explains additional variation in full-scale IQ and is also related to performance IQ and educational attainment. The third factor explains variation in nonword repetition, word reading, and educational attainment. Factors 1 and 3 are highly correlated, indicating that the genetic architecture underlying word reading does not differ much from nonword reading, spelling, and phoneme awareness. Nonword repetition, on the other hand, is genetically more distinct, as indicated by evidence for specific genetic influences not captured by the model. Specific genetic influences were also evident for full-scale IQ and educational attainment. Thus, although reading- and language-related traits show genetic overlaps with full-scale IQ and educational attainment, the model indicates that these traits also have unique unshared components, in line with genetic correlation estimates that are lower than one.

### Limited Evidence for Genes Previously Reported in Reading-/Language-Related Traits and Disorders.

The number of previous GWAS on reading-/language-related traits and disorders is small (Dataset S10), and these have identified very few associations exceeding genome-wide significance. In those prior studies, a total of 48 independent SNPs met a less stringent threshold of *P* < 1 × 10^−6^ in the respective GWAS. We ran lookups of each of those SNPs in our GenLang GWAS meta-analysis results (Dataset S10). Where SNP associations passed a threshold adjusted for multiple testing of 48 SNPs and 2.15 independent GenLang traits (*P* < 4.84 × 10^−4^), we then reran the association analyses after exclusion of the original cohort(s) in which the association was first identified to evaluate independent effects beyond those of the respective earlier study. According to these criteria, only one SNP, rs1555839, previously associated with rapid automatized naming in the Genes, Reading, and Dyslexia (GRaD) cohort (7) yielded a significant signal in the remainder of the GenLang cohorts, showing association with spelling (*P* = 3.33 × 10^−4^). This SNP is one of five SNPs that reached the threshold for genome-wide significance in the original GWAS of the GRaD cohort.

Some 20 genes have been described in the literature as candidate genes for reading-/language-related traits and disorders based on a range of mapping approaches and have been the focus of much of the prior published research in this area (4). Dataset S11 gives gene-based *P* values from our GenLang GWAS meta-analysis, calculated by Multi-marker Analysis of GenoMic Annotation (MAGMA) (22), for each of these genes. Variation in one, namely *DCDC2*, showed association with nonword reading that passed the significance threshold for multiple testing of 20 genes and 2.15 independent GenLang traits (*P* < 0.0012). *DCDC2* was originally identified in a linkage region for dyslexia susceptibility, and SNPs in and near this gene were subsequently associated with dyslexia in candidate gene studies (23), although some investigations, including a meta-analysis of seven studies, failed to support this (24). No single candidate SNP highlighted in prior studies of *DCDC2*, nor in any other candidate gene, was significantly associated with any traits in the GWAS meta-analysis results after correction for testing of 54 SNPs and 2.15 independent GenLang traits (*P* < 4.31 × 10^−4^) (Dataset S11). These null findings highlight the importance of large-scale studies for robust identification of common DNA variation associated with reading-/language-related traits and disorders and warrant a reevaluation of the contributions of candidate genes that are prominent from prior literature.

### A Multivariate GWAS Analysis of GenLang Traits Maximizes SNP Heritability.

To improve the power of our GWAS meta-analysis for follow-up analyses, we took advantage of the high genetic correlations between the traits by performing a multivariate GWAS analysis of word reading, nonword reading, spelling, and phoneme awareness with the Multi Trait Analysis of GWAS (MTAG) method (25). This approach improves the effect estimates of univariate GWAS results per SNP by incorporating information from the other genetically correlated traits. Although MTAG generates output for each primary input trait, these were extremely similar as a consequence of the particularly high genetic correlations between the traits. Hence, the multivariate results for word reading were used for all follow-up analyses because the univariate word reading GWAS meta-analysis has the largest sample size (27,180 for the European ancestry analysis compared with 12,411 to 17,278 for the other three traits). Although no individual SNP reached genome-wide significance in this multivariate analysis (*SI Appendix*, Fig. S4), the approach improved power for follow-up analyses in three ways: 1) by incorporating all available data from the different traits without increasing multiple testing burden; 2) by reducing error variance and thereby, increasing the proportion of phenotypic variability captured by genetic variations, with SNP-based heritability that was higher than any of the univariate estimates with a smaller SE (0.29, SE = 0.02); and 3) because joint analysis of the different traits maximized the effective sample size of the dataset. Indeed, MTAG estimated the GWAS equivalent sample size of the multivariate results as 41,783 compared with 27,180 for the corresponding univariate results. These multivariate GenLang GWAS results were used for follow-up analyses utilizing not only genetic correlation approaches, but also other genome-based methods, such as gene property analysis and heritability partitioning, further explained below.

### Assessing Links to Genetics of Neuroanatomical Variation in Reading-/Language-Related Circuitry.

**Fig. 2.**
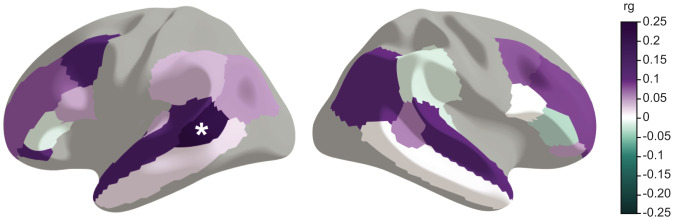
The multivariate GenLang GWAS results show significant genetic correlation with the cortical surface area around the left STS. Genetic correlations (rg) were estimated with LDSC. Included traits are 58 structural brain imaging traits from the UK Biobank selected based on known links of regions and circuits with language processing. The results of the 22 cortical surface areas are shown; gray areas were not included in the analysis. Full results can be found in Dataset S12 and *SI Appendix*, Figs. S6 and S7. *Significant genetic correlation after correcting for 24.85 independent brain imaging traits (*P* < 2.01 × 10^−3^).

The neurobiological circuitry involved in spoken and written language has been extensively investigated in prior literature, from pioneering postmortem studies of brain lesions through to the noninvasive structural and functional neuroimaging research that is now standard for the field (26, 27). Notably, multiple studies have identified relationships between measures of neuroanatomical features and performance on reading-/language-related tasks through analyses of developmental changes, of individual differences, and of relevant disorders (28, 29). At the same time, a growing number of large-scale GWAS efforts have reported robust relationships between common DNA variation and individual differences in multiple aspects of human neuroanatomy, including brain volumes, surface area and thickness of different cortical regions, and white matter microstructure, among others (30, 31). The availability of GWAS data from behavioral/cognitive phenotypes and from magnetic resonance imaging (MRI)-based measures of neuroanatomy makes it possible to determine genetic overlaps between brains and behavior, even when the study cohorts are independent (31, 32). Here, we applied this strategy to assess genetic relationships between reading-/language-related measures and neuroanatomical variation, as follows. First, we performed a literature review to select structural neuroimaging traits that 1) encompass brain regions and white matter tracts with known links to aspects of reading and language and 2) have been investigated with GWAS in the large UK Biobank resource (*Materials and Methods*). This yielded 58 structural neuroimaging traits, including surface-based morphometry (surface area and thickness) (*SI Appendix*, Fig. S6) phenotypes and diffusion tensor imaging (mean and weighted mean fractional anisotropy) (*SI Appendix*, Fig. S7) results. Second, as many of these brain-based phenotypes were significantly correlated with each other, genetic correlations among their summary statistics were used to calculate the number of independent traits for multiple testing correction. Third, we performed genetic correlation analyses between the multivariate GenLang GWAS results and the 58 neuroimaging traits to identify genetic overlaps. One neuroimaging trait showed significant genetic correlation with the multivariate GenLang results (*P* < 0.05/24.85 independent traits = 2.01 × 10^−3^): the surface area of the banks of the superior temporal sulcus (STS) of the left hemisphere (rg = 0.21, SE = 0.06) (Fig. 2 and Dataset S12). This finding suggests the existence of shared genetic factors that contribute both to left STS surface area and to reading-/language-related skills, albeit without identifying which SNPs underlie the relationship. Functional MRI studies have linked this region to different aspects of written and spoken language processing (33–36). To investigate further whether the five different reading- and language-related traits show similar or diverse genetic correlations for banks of the left STS, we went on to specifically assess the results from the original univariate GenLang GWAS meta-analyses, finding consistent genetic correlations (range = 0.18 to 0.23) (Dataset S12).

### Genetic Correlation with Traits from the UK Biobank and Brain-Related Traits from the LD Hub.

**Fig. 3.**
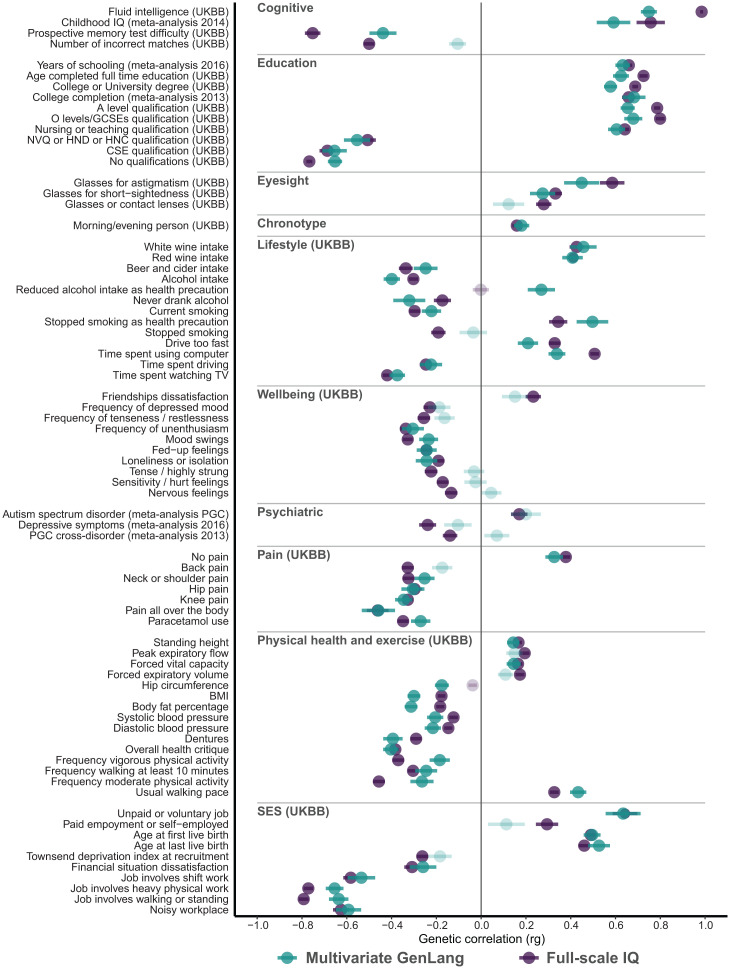
Genetic correlation results of the multivariate GenLang GWAS analysis with comparisons with those for the largest published GWAS of full-scale IQ in LD Hub. Summary statistics for full-scale IQ (*n* = 257,828) were obtained from the Social Science Genetic Association Consortium (18). Genetic correlations between the multivariate GenLang results (blue–green), full-scale IQ (purple), and traits in LD Hub reveal an overlap with cognitive traits, education, eyesight, chronotype, lifestyle, well-being, psychiatric disorders, pain, physical health and exercise, and socioeconomic status. A subset of representative traits is shown; 143 traits showed significant associations with the multivariate GenLang results, and 245 traits showed significant correlations with full-scale IQ, of which 135 traits overlap after correction for multiple testing for 535 × 2 traits (*P* < 4.67 × 10^−5^). Significant correlations are shown in dark colors; nonsignificant correlations are in light colors. Full results can be found in Dataset S13. UKBB: UK Biobank, GCSE: General Certificate of Secondary Education, NVQ: National Vocational Qualification, HND: Higher National Diploma, HNC: Higher National Certificate, CSE: Certificate of Secondary Education, PGC: Psychiatric Genomics Consortium, BMI: body mass index, SES: socioeconomic status. Genetic correlation (rg) is presented as a dot, and error bars indicate the SE.

Next, we assessed genetic correlations of our multivariate GWAS results with 20 cognitive, education, neurological, psychiatric, and sleeping-related traits and 515 additional UK Biobank traits using LD Hub. To further investigate overlaps with and differences from IQ, genetic correlations between these 535 traits and the published GWAS summary statistics for full-scale IQ (18) (*n* = 257,828) were obtained as well. A total of 143 traits showed significant genetic correlations with the multivariate GenLang GWAS results after correction for multiple testing [*P* < 0.05/(535 × 2) = 4.67 × 10^−5^] (Dataset S13), while 245 traits were genetically correlated with full-scale IQ; 135 traits showed significant correlations with both our multivariate GWAS and full-scale IQ. Traits with strong genetic correlations were related to education, eyesight, chronotype, well-being, lifestyle, physical health and exercise, and socioeconomic status. Representative traits are plotted in Fig. 3. Cognitive and education-related traits showed higher genetic correlations with full-scale IQ than with the multivariate GenLang GWAS data. Several psychiatric and well-being traits showed significant (negative) genetic correlations with full-scale IQ but not with our multivariate GWAS: for example, depressive symptoms; cross-disorder susceptibility (from the Psychiatric Genomics Consortium GWAS); and tense, hurt, and nervous feelings. In contrast, several traits related to physical health and lifestyle showed larger genetic correlations with the multivariate GenLang GWAS results than with full-scale IQ, including body mass index, reduced alcohol intake as a health precaution, and usual walking pace.

### Analysis of Genomic Annotations Related to Human Evolution.

Writing and reading are relatively recent cultural innovations, but multiple lines of evidence indicate that the relevant skills, especially those involved in decoding as assessed here, are based on our capacities for spoken language, which emerged through biological evolution along the lineage that led to humans (37, 38). As noted above, our multivariate GWAS captured common DNA variation accounting for a substantial proportion of interindividual variability in reading-/language-related traits within our study populations. We went on to test in aggregate whether there were overlaps between the genomic regions driving this association signal and regions involved in aspects of human evolution over a range of timescales. To do so, we used LDSC heritability partitioning (39), a method that uses GWAS results to investigate whether common DNA variants in a certain set of genomic regions, named an annotation, explain a larger proportion of the SNP-based heritability of the trait than is expected based on the size of that annotation. Building on prior work on evolution of human brain structure (40), we studied five annotations reflecting different aspects of human evolution spanning periods from 30 Mya to 50,000 y ago (*SI Appendix*, Fig. S8). The tested annotations included human gained enhancers active in fetal and adult brain tissue, ancient selective sweep regions, Neanderthal introgressed variants, and archaic introgression deserts (details are in *SI Appendix*, *Extended Methods*). The latter two annotations relate to admixture events between *Homo sapiens* and Neanderthal populations that took place when the different hominins encountered each other outside Africa some 50,000 to 60,000 y ago, with the consequent gene flow leaving remnants (introgressed fragments) that can be detected in the genomes of living humans. Archaic deserts are long stretches in the human genome that, despite these admixture events, are significantly depleted for Neanderthal alleles in living humans, possibly due to critical functions of the genetic loci in *H. sapiens* and intolerance to gene flow (41). We observed significantly enriched heritability for archaic deserts, which was robust to multiple testing correction for analysis of five annotations (*P* < 0.01) (Dataset S14). Thus, our results suggest that common DNA variation in archaic deserts makes a larger contribution to individual differences in reading-/language-related traits within present-day populations than expected by chance.

### Functional Enrichment Using Heritability Partitioning and MAGMA Gene Property Analysis.

We next investigated whether regions of the genome with tissue-specific functions are involved in reading-/language-related traits, again using LDSC heritability partitioning. We used annotations reflecting chromatin signatures from a broad range of tissues, as most variants identified in GWAS are located outside coding regions and are often found enriched in tissue-specific functional regions of the genome, such as promoters, enhancers, and regions with open chromatin. After correction for testing of 489 annotations (*P* < 1.02 × 10^−4^), three annotations showed significant heritability enrichment: histone-3 lysine-4 monomethylation (H3K4me1) in two fetal brain samples and the adult brain germinal matrix (*SI Appendix,* Fig. S9 and Dataset S14). H3K4me1 is considered a marker for enhancer regions. These results indicate that SNPs associated with reading-/language-related traits are overrepresented in fetal brain enhancers.

Next, we used MAGMA gene property analysis (22) to study whether the multivariate GenLang GWAS results were enriched in a specific tissue or brain cell type using tissue-specific and cell type–specific gene expression data in Functional Mapping and Annotation (FUMA) (42, 43). As MAGMA corrects for average expression, each comparison can only answer the question of whether the tissue or cell type is more related to the multivariate GWAS results than the average of the tissues or cell types in the dataset. After correction for 83 tissues (*P* < 6.02 × 10^−4^), no relation was found with tissue-specific gene expression patterns of adult tissues from the Genotype-Tissue Expression (GTEx) project and brain tissues of a specific (developmental) time from Brainspan (Dataset S15 and *SI Appendix*, Fig. S10). In the cell type–specific gene expression analysis, three single-cell RNA-sequencing datasets of embryonic, fetal, and adult brain tissue were used. After correction for 142 cell types (*P* < 3.52 × 10^−4^), the multivariate GWAS results were significantly associated with one of the mature neurons from the fetal dataset: red nucleus neurons (beta = 0.24, SE = 0.07) (Dataset S15 and *SI Appendix*, Fig. S11). This observation could reflect an association with this specific nucleus or with the higher maturity of the neurons compared with the other cell types in the fetal dataset. The red nucleus is a large structure in the ventral midbrain that is part of the olivocerebellar and cerebello–thalamo–cortical systems. It plays roles in locomotion and nonmotor behavior in various animals, and in humans, it might also play a role in higher cortical functions (44).

## Discussion

We performed GWAS meta-analysis of five quantitative reading- and language-related traits (word reading, nonword reading, spelling, phoneme awareness, and nonword repetition) in sample sizes (up to ∼34,000 participants) that are substantially larger than previous genetic analyses of reading and/or language skills assessed with neuropsychological tools (prior GWAS efforts with maximal sizes of *n* = 1,331 to 10,819) (5–7, 45–47). We identified genome-wide significant association for word reading (rs11208009 at chromosome 1, *P* = 1.10 × 10^−8^), highlighting *DOCK7*, *ATG4C*, *ANGPTL3*, and *USP1* as potential candidates for involvement in this trait. Other SNPs from the locus have been associated with triglyceride and cholesterol levels (48) but may represent an independent association signal. Robust SNP-based heritabilities were observed, ranging from 0.13 for nonword repetition to 0.26 for nonword reading. These SNP-based heritabilities are similar in magnitude to those of the related trait dyslexia (estimates range from 0.15 to 0.25 on a liability scale) (49, 50); psychiatric traits, such as Attention Deficit Hyperactivity Disorder symptoms, in adults (0.22) (51); and brain imaging traits, such as cortical surface area (range from 0.12 to 0.33 for different regions) (52) and cortical thickness (range from 0.08 to 0.26) (52). They are larger than that of psychiatric traits, such as major depression (0.08) (53) and alcohol dependence (0.09) (54). So, despite highlighting the need for larger sample sizes to identify loci that individually exceed genome-wide significance, our GenLang GWAS already allowed for multiple informative follow-up analyses based on the full dataset of common variants across the genome, yielding findings that connect to neuroanatomical variation, human evolutionary history, and other aspects of the biology of spoken and written language.

Our work shows overlapping contributions of common genetic variants to individual differences in reading-/language-related and cognitive traits. Such overlaps are evident both from genetic correlation analyses and from the three-factor structural equation model, in which one factor explains most of the variation in word reading, nonword reading, spelling, and phoneme awareness. This is in line with the widespread pleiotropy found between many aspects of cognitive functioning, including language, reading, mathematics, and general cognitive ability (6, 55). We note that summary statistics from the current investigation have also been used to investigate genetic overlaps with self-report of dyslexia diagnosis in an independent GWAS by 23andMe (∼52,000 cases), yielding substantial negative genetic correlations between GenLang quantitative traits and dyslexia status (e.g., −0.71 for word reading, −0.75 for spelling) as reported by Doust et al. (50). Yet, nonword repetition, IQ, and educational attainment have, at least in part, different genetic foundations, as reflected in the residual genetic variation contributing to these traits that is not captured by the model. These findings are consistent with multiple behavioral studies showing a distinction between nonword repetition and other reading- and language-related traits (56). They are also in line with recent structural equation modeling of genetic trait interrelatedness for different reading- and language-related measures in the Avon Longitudinal Study of Parents and Children (ALSPAC) cohort, which demonstrated a shared genetic factor accounting almost fully for the genetic variance in literacy-related phenotypes but for only 53% of that in nonword repetition (57). The 23andMe GWAS on self-reported dyslexia further shows the existence of effects on reading ability that are independent of IQ; of 42 genome-wide significant loci in that study, less than half had previously been associated with cognitive ability or educational attainment in prior high-powered investigations (50). Overall, our work thus enhances understanding of not only the overlaps but also, the distinctions between different reading-/language-related traits and more general cognitive abilities.

Further evidence of differences in trait etiology between the five reading-/language-related traits was observed in our genetic correlation analysis with GWAS results of components (identified via decomposition analysis) from school grades in the Danish iPSYCH cohort (20) and with results of a GWAS by subtraction analysis of educational attainment and cognitive performance (19). Higher scores on phoneme awareness and spelling appear to be genetically correlated with better performance in written than in oral examinations. This may reflect the greater importance of phoneme awareness and spelling for proficient writing than for oral language. Interestingly, word and nonword reading skills were associated with better performance in language than mathematics but not with better performance in written than oral language. Such findings may offer further proof that key reading skills, especially those involved in decoding as assessed in our GWAS meta-analyses, originate from oral language skills (58). “Component 4,” corresponding to relatively better performance in Danish (the native language of the participants in that study) as compared with performance in English, showed negative genetic correlations with our GenLang nonword repetition measure, possibly reflecting the particular importance of verbal short-term memory in second-language learning (59, 60). The results of the GWAS by subtraction, previously proposed to represent so-called “noncognitive” abilities related to educational attainment, such as motivation, curiosity, and persistence, were genetically correlated with word and nonword reading as well as nonword repetition in GenLang. Of note, genetic correlation analyses may be influenced by genetic nurture, the process whereby DNA variants of the parents affect phenotypic outcomes in their children (61). Genetic variants relating to the socioeconomic status of the family may, for example, be involved, as was recently found for cognitive traits (62). Future investigations that include information about nontransmitted alleles (63) and/or data from siblings (62) may help to disentangle pleiotropy from genetic nurture.

Human abilities to process spoken and written language depend on an array of distributed brain circuits (28, 29, 64–66). We performed genetic correlation analyses of our multivariate GenLang GWAS with summary statistics from 58 MRI-based neuroanatomical phenotypes chosen because they concerned brain areas and/or tracts with known links to language processing (28, 29, 65, 66). We identified a significant genetic correlation with cortical surface area of the banks of the STS on the left hemisphere. The STS is a region where the processing of spoken and written language converges, in between modality-specific preprocessing and language comprehension (33–36). A broad range of language-related functions has been previously linked with the left STS through (meta-analyses of) functional MRI and positron emission tomography studies, including those essential for the reading- and language-related traits included in the GWAS meta-analyses: sublexical processing of speech (67, 68) and representation of phonological word forms (69). The importance of this brain area for reading-related traits is also evident from a meta-analysis of structural MRI studies that found lower gray matter volume in the left STS related to reading disability and poor reading comprehension (70). Thus, findings from the genetic correlation analysis are consistent with the role of the STS as a hub where the processing of different language modalities gets integrated as well as the lateralization of such functions. Note, however, that while our work supports the existence of shared genetic factors influencing both left STS surface area and psychometric measures of reading-/language-related skills, it does not inform about potential causal relationships or direction of effects, nor does it identify which particular molecular mechanisms may be involved.

Capacities for acquiring spoken and written language appear unique to our species, building on underlying skills that emerged on the lineage leading to modern humans, but evolutionary accounts remain subject to considerable debate (37, 38). To explore whether GWAS data could give insights in this area, we used heritability partitioning to analyze five annotations representing different time frames and aspects of human evolution. Archaic introgression deserts, defined as genomic regions that are significantly depleted of Neanderthal ancestry, were enriched for genetic variants showing associations in our multivariate GenLang GWAS. Such regions are thought to correspond to genomic loci that were intolerant to the gene flow from Neanderthal populations into *H. sapiens*, which took place through admixture events around 50,000 to 60,000 y ago (41). These loci are enriched for conserved and functional genomic elements: promoters and regions conserved in primates (71) and enhancers active in many tissues as well as those specific for expression in fetal brain and muscle (72). Of all regulatory regions in archaic deserts, brain enhancers show signs of the most stringent purifying selection against introgressed Neanderthal variation. This evidence of conservation and selection indicates that archaic deserts mark parts of the genome where variation has a high probability of deleterious consequence (72). Enriched heritability for reading-/language-related traits in archaic deserts seems broadly consistent with differences between Neanderthals and *H. sapiens* in evolutionary trajectories for language emergence. However, analyses of this kind are only indirectly informative and cannot pinpoint any specific time frame of human evolution during which genetic variants associated with reading- and language-related traits were introduced. Investigations that further integrate data on reading-/language-related genetic signals and evolutionary annotations of hominin genomes would be warranted to shed further light on these complex questions.

Regarding functional implications of our findings, heritability partitioning of the multivariate GenLang GWAS results identified enrichment in enhancer regions present in fetal brain tissue and the adult germinal matrix. The enhancer regions of the germinal matrix are highly similar to those of fetal brain tissues and not the other adult brain tissues we studied, likely reflecting the neural stem cell population present in that tissue (73). In these analyses, there was no specific association with one particular brain cell type. However, follow-up work with MAGMA using single-cell RNA-sequencing data from fetal, embryonic, and adult brain uncovered a significant association with fetal neurons from the red nucleus, which may relate to the more adult state of these neurons compared with the other cell types in the fetal dataset (74). The MAGMA analysis could not be used to test for replication of the association with (fetal) brain tissue of the LDSC heritability partitioning, as results in this case are corrected for the association with the average expression of the dataset (22, 42), and the datasets with fetal data only included brain samples.

Our findings prompt a major reevaluation of prior literature on genetic associations with reading and language traits, especially with regard to candidate gene studies. The large-scale GWAS meta-analysis results made it possible to robustly and systematically investigate evidence for association of previously reported candidate SNPs/genes and suggestive genome-wide screening results from prior studies of reading-/language-related traits and disorders. Of the 54 candidate SNPs and 20 candidate genes that we assessed (none of which met genome-wide significance), only *DCDC2* yielded an association that survived correction for multiple testing in the context of targeted replications. This locus showed association only at the gene-based level and with one trait: nonword reading. Some previously reported associations in the literature could reflect the specific language, phenotype, or recruitment procedure of the cohort in which the gene or variant was investigated and/or differences between contributions of common and rare variation at a locus of interest. Yet, the lack of support here also suggests that false-positive results have made an impact on the field, most likely related to limited sample size in prior reports, which is known to elevate the risk of type 1 error (75). Regarding validation of findings from the previous more limited GWAS of quantitative reading-/language-related traits, our large-scale meta-analysis identified association with spelling for the SNP rs1555839, previously found to be associated with rapid automatized naming and rapid alternating stimulus (7). Overall, these results highlight the need for a genome-wide perspective and the importance of large well-powered samples if we are to obtain reliable insights into the role of common genetic variants in language- and reading-related traits.

Reading- and language-related phenotypes pose special challenges for scaling up genetic analysis since psychometric assessments can be labor intensive to administer and score and because of the heterogeneity introduced by differences in assessment tools, ages, populations, and languages, among other factors (2, 76). One-item questions have enabled increases in sample size for GWAS of a wide range of traits and disorders, especially when available through large resources, such as the UK Biobank. As discussed above, a single self-report item about dyslexia diagnosis enabled an informative GWAS in the 23andMe research cohort (50). However, no validated questions have yet been described that adequately capture interindividual variability in reading and language skills in the normal range, which still requires administration of psychometric tests. The GenLang Consortium was established as an international effort by multiple research teams with the aims of overcoming such difficulties through a range of strategies and enabling large-scale well-powered investigations of genomic underpinnings of these important traits.

This first wave of analysis from GenLang represents the largest GWAS meta-analyses for direct quantitative assessments of reading- and language-related abilities to date, including 22 cohorts with data available for at least one of the phenotypes. Nonetheless, although substantially increased over prior work in this area, sample sizes may still be considered relatively modest compared with the state of the art for genetic association analyses of other complex traits. While they captured a significant proportion of the genetic variation underlying each phenotype, yielding several insights into the associated biology, detection of individual genome-wide significant loci was still limited. In addition, when sufficiently large datasets become available, it would be valuable to validate the genetic correlation and heritability partitioning results in additional independent datasets. A number of phenotypes of interest, including those that tap into syntactic skills or that involve different modalities such as sign languages, could not (yet) be pursued due to inadequate sample sizes, even when combining data available from multiple cohorts. We note that despite our best efforts at harmonizing the included datasets and limited evidence of heterogeneity in the results based on Cochran Q statistics, LDSC intercepts, and genetic correlations between subsets of the data, we cannot fully exclude that heterogeneity is introduced by the inclusion of data from different assessment tools, languages, and ages. The choice of assessment tools for future collection of reading- and language-related phenotypes for genomic studies, to increase the sample sizes of these GWAS meta-analyses and also, to collect additional language-related phenotypes, should, therefore, be based at least partially on optimal matching with existing data. At the same time, we should invest in facilitating and simplifying the collection of language-related phenotypes, in part by developing and optimizing test batteries that could be reliably administered online in web/app-based settings. These are major areas of focus for the GenLang Consortium moving forward.

In summary, our GWAS meta-analyses of five reading- and language-related phenotypes in sample sizes of up to ∼34,000 participants demonstrated significant SNP heritability for all traits and identified genome-wide significant associations with word reading on chromosome 1. Structural equation models revealed a single factor accounting for much of the genetic architecture underlying word reading, nonword reading, spelling, and phoneme awareness, prompting a multivariate GWAS analysis. The multivariate results were genetically correlated with cortical surface area of the banks of the left STS, a brain region where the processing of spoken and written language comes together. Partitioned heritability analyses showed enrichments in fetal brain enhancers, highlighting links to early brain development, and in archaic deserts depleted of Neanderthal ancestry, suggesting that genomic regions associated with emerging language-related skills in *H. sapiens* may have been intolerant to gene flow from other archaic hominins. These efforts by GenLang open up avenues for deciphering the biological underpinnings of spoken and written language.

## Materials and Methods

A more detailed version of the methods is provided in *SI Appendix*, *Extended Methods*.

### Study Cohorts.

The meta-analyses included GWAS summary statistics from 22 independent cohorts (Dataset S1). Different measures for the reading- and language-related traits had been assessed in each cohort and could be included in the GWAS meta-analyses (*SI Appendix*, *Supplemental Notes* has details of each measure, and Dataset S1 has an overview of the included measures and sample sizes for each cohort). Data from children, adolescents, and young adults aged 5 to 26 y were included. The phenotype data were standardized and then, adjusted for covariates (age, age^2^, sex, and ancestry principal components [PCs]); the genotype data were subjected to stringent quality control according to a detailed analysis plan following standard procedures for GWAS and then imputed using the Haplotype Reference Consortium version 1.1 panel or the 1000 Genomes Project Phase 3 reference panel (Dataset S2). Univariate GWAS analyses were performed using linear regression methods with the imputed additive genotype dosages for the full dataset and for males and females separately.

### Meta-Analyses.

The summary statistics for each GWAS cohort for each trait were subjected to stringent quality control measures (SNPs were excluded if imputation quality scores were <0.7, minor allele frequency was <0.01, and/or minor allele count was ≤10). Meta-analyses of the univariate GWAS results were performed with METAL (version March 2011), with effect size estimates weighted using the inverse of the corresponding SEs. For the follow-up analyses with LDSC, separate meta-analyses without genomic control correction (77) were performed using only individuals of European ancestry as determined by ancestry PCs. To accommodate the multiple testing burden present in performing separate meta-analyses for the five reading- and language-related traits while taking into account the high phenotypic correlations between them, we calculated the effective number of independent variables (VeffLi) from the meta-analysis results using PhenoSpD (78) (v1.0.0). The Bonferroni-corrected genome-wide significant *P* value threshold was determined at 2.33 × 10^−8^ (5 × 10^−8^/2.15 independent traits).

### GenomicSEM.

To investigate the high genetic correlations between the summary statistics of the five reading- and language-related traits and with cognitive performance (18) and educational attainment (18), we used GenomicSEM (version 0.03) (21) to model the joint genetic architecture. We used a two-step approach. First, exploratory factor analysis for models with one to four factors was run; second, the model that explained the largest part of the variance in data was chosen for follow-up with confirmatory factor analysis in GenomicSEM. Different models were tested based on the exploratory three-factor model, with different factor loadings based on strength thresholds between 0.1 and 0.5, and the optimal model was chosen based on several indices of model fit.

### Multivariate GWAS Analysis.

A multivariate GWAS was performed on the univariate GWAS summary statistics of the four traits with the highest genetic correlations (word reading, nonword reading, spelling, and phoneme awareness) using MTAG (v1.0.8) (25).

### Heritability and Genetic Correlation.

LDSC (77) (v1.0.0) was used to estimate genomic inflation and SNP-based heritability of the meta-analysis results and to investigate genetic correlations (13). All analyses were based on HapMap 3 SNPs only, and precalculated LD scores from the European 1000 Genomes reference cohort were used. GWAS summary statistics for genetic correlation analyses with cognitive traits were obtained from the Social Science Genetic Association Consortium (18) and the GWAS catalog (79) and through collaboration with the iPSYCH Consortium (20). Publicly available GWAS summary statistics of neuroimaging traits were obtained via the Oxford Brain Imaging Genetics Server (30); a total of 58 neuroanatomical phenotypes were selected based on their relevance to language processing. PhenoSpD (78) was used to calculate the effective number of independent variables (VeffLi) to inform the multiple testing correction of the genetic correlation analyses with the cognitive and brain imaging phenotypes. Following our targeted analysis of brain imaging traits, genetic correlations were estimated between the MTAG results and summary statistics of 20 cognitive, education, neurological, psychiatric, and sleeping-related traits and all 515 UK Biobank traits available in LD Hub (80) (v1.9.3).

### Functional Mapping and Annotation of GWAS Meta-Analysis Results.

The FUMA platform (version 1.3.6a) (43) was used to annotate the genome-wide significant variants.

### Gene and Gene-Set Analysis.

MAGMA (22) (version 1.08) gene analysis in FUMA was used to calculate gene-based *P* values and for gene-property analyses, to study relationships with tissue-specific and cell type–specific gene expression patterns. Several bulk RNA-sequencing and single-cell RNA-sequencing datasets of brain samples were assessed through FUMA (43).

### Partitioning Heritability of Chromatin and Evolutionary Signatures.

LDSC heritability partitioning (39) was used to estimate the enrichment of heritability of the MTAG results in annotations reflecting evolutionary signatures from different periods along the lineage leading to modern humans, ranging from around 50,000 y ago back to 30 Mya [adapting a pipeline recently published by Tilot et al. (40)]. In addition, LDSC heritability partitioning was used to study the association with several annotations reflecting tissue-specific chromatin modification patterns. Annotations were based on data from the Roadmap Epigenomics project and Enhancing GTEx project (ENTEx) processed by Finucane et al. (81).

## Supplementary Material

Supplementary File

Supplementary File

Supplementary File

Supplementary File

Supplementary File

Supplementary File

Supplementary File

Supplementary File

Supplementary File

Supplementary File

Supplementary File

Supplementary File

Supplementary File

Supplementary File

Supplementary File

Supplementary File

## Data Availability

The full GWAS summary statistics (for word reading, nonword reading, spelling, phoneme awareness, nonword repetition, performance IQ, each available as "full" and "European subset" datasets) are available through the GWAS Catalog (accession nos. GCST90104462–GCST90104472) and the website of the GenLang network (http://www.genlang.org/) (82, 83). Code used to perform the meta-analysis and follow-up analyses is available in GitLab (https://gitlab.gwdg.de/else.eising/genlang_quantitative_trait_gwasma) (84).
