# Supplementary material for: Genome-wide analyses of individual differences in quantitatively assessed reading- and language-related skills in up to 34,000 people
Source: Proc Natl Acad Sci U S A. 2022 Aug 23;119(35):e2202764119. doi: 10.1073/pnas.2202764119 (PMC9436320; doi:10.1073/pnas.2202764119)
Supplement: Supplementary File [file pnas.2202764119.sapp.pdf]

PNAS
www.pnas.org

Supplementary Information for

Genome-wide analyses of individual differences in quantitatively assessed reading- and language-related skills in up to 34,000 people

Else Eising, Nazanin Mirza-Schreiber, Eveline L. de Zeeuw, Carol A. Wang, Dongnhu T. Truong, Andrea G. Allegrini, Chin Yang Shapland, Gu Zhu, Karen G. Wigg, Margot L. Gerritse, Barbara Molz, Gökberk Alagöz, Alessandro Gialluisi, Filippo Abbondanza, Kaili Rimfeld, Marjolein van Donkelaar, Zhijie Liao, Philip R. Jansen, Till F. M. Andlauer, Timothy C. Bates, Manon Bernard, Kirsten Blokland, Milene Bonte, Anders D. Børglum, Thomas Bourgeron, Daniel Brandeis, Fabiola Ceroni, Valeria Csépe, Philip S. Dale, Peter F. de Jong, John C. DeFries, Jean-François Démonet, Ditte Demontis, Yu Feng, Scott D. Gordon, Sharon L. Guger, Marianna E. Hayiou-Thomas, Juan A. Hernández-Cabrera, Jouke-Jan Hottenga, Charles Hulme, Juha Kere, Elizabeth N. Kerr, Tanner Koomar, Karin Landerl, Gabriel T. Leonard, Maureen W. Lovett, Heikki Lyytinen, Nicholas G. Martin, Angela Martinelli, Urs Maurer, Jacob J. Michaelson, Kristina Moll, Anthony P. Monaco, Angela T. Morgan, Markus M. Nöthen, Zdenka Pausova, Craig E. Pennell, Bruce F Pennington, Kaitlyn M. Price, Veera M. Rajagopal, Franck Ramus, Louis Richer, Nuala H. Simpson, Shelley D. Smith, Margaret J. Snowling, John Stein, Lisa J. Strug, Joel B. Talcott, Henning Tiemeier, Marc P. van der Schroeff, Ellen Verhoef, Kate E. Watkins, Margaret Wilkinson, Margaret J. Wright, Cathy L. Barr, Dorret I. Boomsma, Manuel Carreiras, Marie-Christine J. Franken, Jeffrey R. Gruen, Michelle Luciano, Bertram Müller-Myhsok, Dianne F. Newbury, Richard K. Olson, Silvia Paracchini, Tomáš Paus, Robert Plomin, Sheena Reilly, Gerd Schulte-Körne, J. Bruce Tomblin, Elsje van Bergen, Andrew J.O. Whitehouse, Erik G. Willcutt, Beate St Pourcain, Clyde Francks, Simon E. Fisher*.

*Simon E. Fisher, Language and Genetics Department, Max Planck Institute for Psycholinguistics, Wundtlaan 1, 6525 XD Nijmegen, The Netherlands. Tel: +31 24 3521441, Fax: +31 24 3521213
Email: Simon.Fisher@mpi.nl.

This PDF file includes:

Extended Methods
Supplementary Notes
Figures S1 to S11
SI References

Other supplementary materials for this manuscript include the following:

Dataset S1 to S15

Extended Methods

Study cohorts

The meta-analyses included GWAS summary statistics from 22 independent cohorts. These were, alphabetically, the Adolescent Brain Cognitive Development[SM] Study (ABCD Study), the Avon Longitudinal Study of Parents and Children (ALSPAC), the ASTON cohort, the Brisbane Adolescent Twin Sample (BATS), the Basque Center on Cognition, Brain and Language (BCBL) cohort, the Colorado Learning Disabilities Research Center (CLDRC) cohort, the Early Language in Victoria Study (ELVS), the Familial Influences on Literacy Abilities (FIOLA) project, Generation R (GenR), the Genes, Reading, and Dyslexia (GRaD) study, the Iowa study, the NeuroDys cohort, the Netherlands Twin Register (NTR), the Pediatric Imaging, Neurocognition, and Genetics (PING) cohort, the Philadelphia Neurodevelopmental Cohort (PNC), the Raine Study, the SLI Consortium (SLIC) cohort, the Saguenay Youth Study (SYS), the Twins Early Development Study (TEDS), the Toronto cohort, the Oxford Dyslexia cohort (UKDYS), and the York cohort (see *Dataset S1* for demographic characteristics for each cohort). The cohorts were collected in different countries: in the USA, UK, the Netherlands, Australia, Canada, Spain, Austria, Germany, Switzerland, Finland, Hungary and France (ordered by sample size). Most participants are therefore from countries with English as their main language. Other languages spoken by participants are Dutch (n≤2,865, depending on trait), Spanish (n≤1,236), German (n≤1,227), Finnish (n≤323), French (n≤137) and Hungarian (n≤225). Most cohorts mainly include participants of European ancestry, with the exception of the GRaD cohort, which consists of individuals of African-American and Hispanic ancestry, and the ABCD Study, GenR and PING cohort, which include multiple ancestries. The sample sizes per cohort range from 104 to 10,187 participants (104 to 5,080 participants of European ancestry, defined by principal component analyses (PCA)). For each cohort, an Institutional Review Board (IRB) or ethical committee approved the respective studies, and participants provided informed consent.

Different measures for the reading- and language-related traits had been assessed in each cohort and could be included in the GWAS meta-analyses (see *SI Appendix,* Notes for details of each measure, and *Dataset S1* for an overview of the included measures and sample sizes for each cohort). Data from children, adolescents and young adults were included in the meta-analysis (age at time of assessment ranging from 5 years to 26 years). Outlier samples, based on the phenotype data (>4 SD from the mean), were removed for each phenotype separately. The phenotype data were then adjusted for covariates (age, age^2, sex and ancestry principal components; age-normed phenotypes were not adjusted for age and age^2; the number of ancestry principal components to be included were determined independently for each cohort by the analyst of that cohort). See *Dataset S2* for details on the included covariates per cohort. Phenotype data for word reading, nonword repetition and performance IQ were standardized to z-scores. Phenotype data for spelling, phoneme awareness and nonword reading were rank transformed to acquire normally distributed data for all cohorts. For follow-up analyses involving multiple phenotypes - the genetic correlation analysis with LDSC (1) and the multivariate GWAS analysis with MTAG (2) - a separate rank transformation was performed for word reading, nonword repetition and performance IQ to further harmonize the phenotype data processing. For male- and female-only association analyses, phenotype data were filtered, adjusted and transformed for male and female subsets separately.

The genotype data were subjected to stringent quality control according to a detailed analysis plan following standard procedures for GWAS, including SNP filters for minor allele frequency, call rate and Hardy-Weinberg equilibrium and sample filters including missingness and (for cohorts of unrelated individuals) relatedness. Cohort-specific details on quality control can be found in *Dataset S2*. Individuals of European ancestry were identified using PCA-based analysis of genetic diversity. Individuals with non-European ancestry were excluded from all cohorts, with exception of ABCD, GenR, GRaD and PING. For the ABCD, GenR and PING cohorts, two association analyses were performed, one including and one excluding individuals of non-European ancestry. Results of datasets including individuals of non-European ancestry were excluded from follow-up analyses with LDSC (1), MAGMA (3) and MTAG (2), since these methods utilize LD information based on

European-ancestry reference data when raw genotyping data are not available (as is the case for this GWAS meta-analysis). The X chromosome was included by all cohorts except for NeuroDys. Genotype data were imputed using the Haplotype Reference Consortium version 1.1 panel for 20 out of the 22 cohorts, and using the 1000 Genomes Project Phase 3 reference panel for the GRaD and SYS cohorts. Single variant association analyses were performed using linear regression methods with the imputed additive genotype dosages for the full dataset, and for males and females separately. For the X chromosome, males were treated as homozygous diploids. Descriptions for each cohort of the samples, phenotype measures, genotyping, quality control and analysis procedures can be found in *Dataset S1 and S2*.

Meta-analyses

The summary statistics for each GWAS cohort for each trait were subjected to stringent quality control measures. SNPs were excluded from the meta-analyses based on low imputation quality scores <0.7, minor allele frequency <0.01 and minor allele count ≤10. Additional quality control of each summary statistics file was performed with EasyQC (4).

Meta-analyses of the summary statistics were performed with METAL (5) (version March 2011), with effect size estimates weighted using the inverse of the corresponding standard errors. A total of 13,633 to 33,959 individuals (12,411 to 27,180 individuals of PCA-selected European ancestry) of 10 to 19 cohorts (no trait was available from all 22 cohorts) were included in the GWAS meta-analyses for the different traits (Table 1 and *Dataset S1*). SNPs for which data were available from less than 5,000 individuals were excluded from the meta-analysis results. The number of SNPs per cohort and for each meta-analysis are provided in (*Dataset S3*). For the heritability and genetic correlation analyses with LDSC, separate meta-analyses without genomic control correction were performed, because the LDSC regression intercept can be used to estimate a more powerful and accurate correction factor than genomic control (1). Only data of individuals of the PCA-selected European ancestry subgroup were included, to allow use of pre-computed LD scores. Computation of LD-scores for the full partially admixed dataset was not possible, since genotyping data of all cohorts were not available at a single site.

To accommodate the multiple-testing burden present in performing separate meta-analyses for the five reading- and language-related traits, while taking into account the high phenotypic correlations between them, we calculated the effective number of independent variables (VeffLi) from the meta-analysis results using PhenoSpD (6) (v1.0.0). The Bonferroni-corrected genome-wide significant P-value threshold was determined at 2.33×10^{-8} (5×10^{-8} / 2.15 independent traits).

We investigated the degree to which differences between cohorts in age distribution and phenotyping tools introduced heterogeneity in the meta-analysis results. First, Cochran's Q test statistics, which assess whether estimated effect sizes are homogeneous across studies, were obtained with METAL, visualized with quantile-quantile plots (Fig. S3) and used to decide between a fixed-effects and random-effects meta-analysis. Based on these analyses, a fixed-effects meta-analysis was performed for all traits except for nonword repetition. Second, LDSC intercept and ratio were inspected to distinguish polygenicity from confounders (1). Third, meta-analyses of subsets of the cohorts were performed, split up by mean age or the type of reading test applied. Heterogeneity caused by difference in mean age and type of reading test was studied by calculating genetic correlations between data subsets using LDSC (7). In addition, meta-analyses for male- and female-only subsets of the data were run as sensitivity analyses, to investigate the degree to which males and females might show differences in SNP-heritability for these traits and show genetic overlap as calculated with genetic correlation analyses.

GenomicSEM

To investigate the high genetic correlations between the reading- and language-related traits, and with cognitive performance and educational attainment, we used genomic structural equation modeling (GenomicSEM; version 0.03) (8) to model the joint genetic architecture. Summary statistics of the five GenLang traits and performance IQ, and published GWAS summary statistics for cognitive performance and educational attainment from the Social Science Genetic Association

Consortium (https://www.thessgac.org/data) (9) were used as input. GenomicSEM first runs multivariable LDSC to obtain genetic covariance and sampling covariance matrices. Next, exploratory factor analyses were run using a maximum-likelihood factor analysis, for models with one to four factors. The exploratory model that explained the largest part of the variance in the data was chosen for follow-up with confirmatory factor analysis in GenomicSEM. Different models were tested based on the exploratory three-factor model, with different factor loadings based on strength thresholds between 0.1 and 0.5. The optimal model was chosen using the following model fit indices: the p-value of the chi-square test, Akaike Information Criterion (AIC), Comparative Fit Index (CFI), and Standardized Root Mean Square Residual (SRMS). The model with the highest p-value, lowest AIC, CFI >0.9, and SRMR <0.1, was considered the best fitting model. (A p-value above 0.05 may not be possible when including summary statistics of large samples (8)). The best fitting model included the factor loadings with a strength ≥ 0.179 from the exploratory model.

Multivariate GWAS analysis

A multivariate GWAS was performed on the four most highly correlated traits: word reading, nonword reading, spelling and phoneme awareness, using Multi Trait Analysis of GWAS (MTAG, v1.0.8) (2), to maximize information for follow-up analyses on biological pathways, evolutionary significance, and so on. MTAG can perform a multivariate GWAS using summary statistics of different but related traits, while correcting for overlapping samples. Because MTAG takes its sample overlap estimates from LDSC (1), univariate meta-analysis results including only individuals of the PCA-selected European subgroup were used. MTAG outputs multivariate results for each input trait, containing effect sizes and p-values for each SNP present in the input files. The MTAG results generated for the four traits were extremely similar as a consequence of the high genetic correlations between the traits, as determined by visual inspection of Manhattan plots and genetic correlation analysis (rg= 1.00, se=5.64×10^{-5} to 3.0×10^{-4} for all comparisons). We therefore took the MTAG results from the multivariate word reading analyses as our dataset for follow-up, since that GWAS had the largest sample size and thus made maximal use of the available information.

Heritability and genetic correlation

LDSC (1) (v1.0.0) was used to estimate genomic inflation and SNP-based heritability of the meta-analysis results, and to investigate genetic correlations (7). All analyses were based on HapMap 3 SNPs only, and precalculated LD scores from the European 1000 Genomes reference cohort were used. For the LDSC analyses of the MTAG results, the GWAS equivalent sample size, estimated by MTAG, was used as sample size. The influence of confounding factors was tested by comparing the estimated intercept of LDSC to one, and the ratio of LDSC to zero. This ratio estimates the proportion of inflation in χ^2 attributable to confounding, as opposed to true polygenic effects. SNP heritability was estimated based on the slope of the LDSC.

GWAS summary statistics for genetic correlation analyses with cognitive traits were obtained from the Social Science Genetic Association Consortium (full-scale IQ and educational attainment(9); https://www.thessgac.org/data); the GWAS catalogue (noncognitive skills investigated with GWAS by subtraction (10); ftp://ftp.ebi.ac.uk/pub/databases/gwas/summary_statistics/GCST90011874), and through collaboration with the iPSYCH consortium (GWAS analysis of Danish school grades (11)). PhenoSpD (6) was used to calculate the effective number of independent variables (VeffLi) to inform the multiple testing correction. A total of 18.28 independent comparisons were performed in Figure 1, the p-value threshold is therefore set to $p=2.74*10^{-3}$.

Publicly available GWAS summary statistics of neuroimaging traits were obtained via the Oxford Brain Imaging Genetics Server (12) (http://big.stats.ox.ac.uk/). Out of 3,144 brain imaging-derived traits with summary statistics available from the UK Biobank, a total of 58 neuroanatomical phenotypes were selected based on their relevance to language processing. Brain imaging traits encompassed surface-based morphometric (SBM) and diffusion tensor imaging (DTI) phenotypes. For SBM, data were originally generated with Freesurfer by parcellation of the white surface (the surface area between the white and grey matter) using the Desikan-Killiany atlas. Both cortical surface area and mean cortical thickness were selected for brain areas that overlapped regions previously related to language processing, based on literature review (13-16). For DTI, tracts

spanning the extended language network (17) were selected, and fractional anisotropy values derived from both tract-based-spatial statistics and probabilistic tractography were used (both mean and weighted-mean fractional anisotropy). Again, PhenoSpd was used to calculate the effective number of independent comparisons. A total of 24.85 independent brain imaging-derived traits were identified. Therefore, the p-value threshold for a significant genetic correlation between the brain imaging-derived traits and the MTAG results was set to $p=2.01 \times 10^{-3}$ (0.05/24.85). In addition to our targeted analysis of brain imaging traits, genetic correlations were estimated between the MTAG results and summary statistics of 20 cognitive, education, neurological, psychiatric and sleeping-related traits and all 515 UK Biobank traits available in LD Hub (18) (v1.9.3, http://ldsc.broadinstitute.org/ldhub/). These 535 traits comprise all phenotypes available through LDhub with relevance to brain function (beyond neuroimaging traits), and all available traits from the UK Biobank. Genetic correlations between these 535 traits and the published GWAS summary statistics for full-scale IQ (9) were obtained as well. The Bonferroni corrected p-value threshold for significance of the LDhub results was $0.05 / (535*2) = 4.67 \times 10^{-5}$. Genetic correlations may reflect pleiotropy, correlation between causal loci or spurious associations, and can inform about shared biological mechanisms and causal relationships between traits (19).

Functional Mapping and Annotation of GWAS meta-analysis results
The platform Functional Mapping and Annotation of Genome-Wide Association Studies (20) (FUMA GWAS; https://fuma.ctglab.nl/; version 1.3.6a) was used to annotate the genome-wide significant variants and to calculate gene-based p-values. Using the SNP2GENE function, genome-wide significant loci were annotated with expression quantitative trait locus (eQTL) data from 4 different databases: GTEx V8 (brain samples only; http://www.gtexportal.org/home/datasets), the blood eQTL browser (http://genenetwork.nl/bloodeqtlbrowser/), the BIOS QTL browser (http://genenetwork.nl/biosqtlbrowser/), and BRAINEAC (http://www.braineac.org/). Loci were also annotated with information on previously associated traits from the GWAS catalog (https://www.ebi.ac.uk/gwas/).

Gene and gene-set analysis
MAGMA (3) (version 1.08) gene analysis in FUMA was used to calculate gene-based p-values from SNPs located in the body of the gene and in the region 1kb upstream to include SNPs located in the promoter region. Look-ups were performed for candidate genes proposed in prior literature on reading-, speech- and language-related traits and disorders. MAGMA accounts for gene-size, number of SNPs in a gene and LD between markers. The gene-based analysis was performed with default parameters (SNP-wide mean model), with the European 1000 Genomes reference cohort phase 3 as reference panel.

The MTAG results were further analysed using MAGMA gene property analyses, to study relationships with tissue-specific and cell type-specific gene expression patterns. Bulk RNA-sequencing data from GTEx V8 (http://www.gtexportal.org/home/datasets) and Brainspan (http://www.brainspan.org) of adult tissue samples and developmental brain samples were assessed in the SNP2GENE analysis in FUMA (20). In addition, single-cell RNA-sequencing data from human embryonic (midbrain, 6-11 weeks post conception; Gene expression omnibus (GEO) accession number GSE76381), fetal (prefrontal cortex, 8-26 weeks post conception; GEO accession number GSE104276) and adult (Allen Brain Atlas cell types data of the middle temporal gyrus; http://celltypes.brain-map.org/) brain samples were used in the Cell Type analysis in FUMA (21). Datasets with brain tissues and cell types were selected based on their availability in FUMA and on being informative of different developmental timeframes. In each dataset, MAGMA performs a one-sided test for each tissue or cell type which essentially assesses the positive relationship between tissue/cell type expression levels and genetic association of genes. Each result in a dataset is then conditioned on the association with the average gene expression pattern of that dataset. Because each dataset is analysed individually, batch effects were avoided that might arise from integration of different datasets. Bonferroni correction was subsequently performed for all bulk RNA-sequencing datasets together (83 tissues), and for all single-cell RNA-sequencing datasets together (142 cell types).

Partitioning heritability of chromatin and evolutionary signatures

LDSC heritability partitioning (22) was used to estimate the enrichment of heritability of the MTAG results in annotations reflecting tissue-specific chromatin modification patterns. Annotations were based on data from the Roadmap Epigenomics project and ENTEX, processed by Finucane et al. (23).

In addition, LDSC heritability partitioning was used to study the association with several annotations reflecting evolutionary signatures and annotations from different periods along the lineage leading to modern humans, ranging from around 50,000 years ago back to 30 million years, adapting a pipeline recently published by Tilot et al. (24). The following annotations reflecting evolutionary features were used:

- Human Gained Enhancers are regulatory regions which are active in human adult or fetal tissues and not active in macaques or chimpanzees. Their activity was determined by chromatin immunoprecipitation followed by sequencing of the histone modification histone-3, lysine-27 acetylation (H3K27ac) and histone-3 lysine-4 dimethylation (H3K4me2), markers of active promoters and enhancers. Thus, these regions gained regulatory function along the lineage that led to our species after our last common ancestor with Old World monkeys some 30 million years ago, and hence might be involved in the emergence of human-specific traits (25, 26).
- Ancient selective sweep regions consist of regions harbouring haplotypes which rose in frequency in the modern human lineage due to an advantageous allele within the haplotype in the last 300-600 thousand years (27).
- Neanderthal-introgressed regions are the genomic variants which were introduced to the human genome by the admixture of Homo sapiens and Neanderthal populations around 50 thousand years (28).
- Neanderthal-introgressed variants are not evenly spread across the genome. Archaic introgression deserts are large stretches of the human genome that are depleted for Neanderthal ancestry, possibly due to critical functions and intolerance to gene flow (29).

These five evolutionary annotations were based on the annotations used previously by Tilot et al. (24) in a study of cortical surface area. We further refined those annotations by removing overlapping genomic regions among annotations. For example, we detected a small number of Neanderthal introgressed SNPs within archaic desert regions, and removed these SNPs from both annotations. Further, we merged four annotations of human gained enhancers active in human foetal cortex at consecutive developmental stages. Ancient selective sweeps and adult brain-tissue expressed HGEs annotations were no different from the prior study.

Supplemental Notes

Phenotype information

A short survey in the GenLang network (www.genlang.org) was used to identify language- and reading-related phenotypes with prior collected data available from multiple cohorts, towards maximizing power for potential meta-analysis. The largest number of cohorts had data on word reading, nonword reading, spelling, phoneme awareness and nonword repetition. A nonword is a combination of letters that looks like a word and follows the phonotactic rules of the language, but has no meaning. In addition, performance IQ (as an index of nonverbal abilities) was analysed.

Cohort inclusion

Cohorts with language-/reading-related phenotypes and matching genome-wide genotype data were invited to join the GWAS meta-analysis effort through the GenLang network, an international consortium of researchers interested in the genomics of speech, language and reading traits. In addition, we identified a number of public cohorts with relevant phenotype and genotype data that had already been made freely available to the research community. Any cohort with measures of word reading accuracy, nonword reading accuracy, spelling accuracy, phoneme awareness and/or nonword repetition accuracy that matched the general description of these phenotypes as stated in the Methods section of the main manuscript, was invited to participate in the GWAS study. The cohorts are from Europe, North America and Australia, and include population-based, twin and disorder-oriented samples. Most cohorts were recruited in countries with English as the major native language, others are from Dutch-, Spanish-, German-, French-, Hungarian- and Finnish-speaking countries. All included phenotypes were assessed in the native language of the participants. *Dataset S1* provides an overview of the cohorts.

First, information from each cohort was collected on the exact phenotype measures available, including the test instrument, derived measures available, sample size, phenotype data distribution and distribution of participant age at time of data collection. A number of cohorts had measures of multiple language- and reading-related traits, sometimes measured with multiple instruments or at multiple ages. When data of multiple instruments or multiple ages were available, the instrument and/or age was used that best matched the instruments and ages collected by the other cohorts. The phenotype data distribution of all included cohorts was used to determine the phenotype data normalization method for each included trait (z-scores or rank transformation). Below you can find a description of the phenotypes included for each cohort; an overview is included in *Dataset S1*.

Individuals over 18 years of age were excluded from all but three cohorts: the BATS cohort, PING and PNC. The PING and PNC cohorts include individuals with a wide age range: 5-21 and 8-22 years of age, respectively. The BATS cohort is a slightly older cohort, including individuals aged 11 to 26. For the BATS cohort, summary statistics were obtained as well for a subset of the cohort aged less than 18 years. Because the inclusion of the full BATS cohort did not lead to additional heterogeneity, compared to including the subset aged up to 18 years of age, the data of the full BATS cohort was used for the GWAS meta-analyses.

Heterogeneity analysis

We evaluated whether heterogeneity related to age and/or use of different test instruments remained in our data. To do so, we generated GWAS meta-analysis results for word and nonword reading, stratified by age or test instrument (*Dataset S4*). We could then use those data to test for genetic correlations, determining to what extent the same common genetic variation is accounting for phenotypic variability in the different stratified GWAS datasets. In relation to age, we compared cohorts with mean age <12 years versus ≥ 12 years (a threshold chosen to yield similarly sized subsets; see *Dataset S1 and S4*). Genetic correlations between the age-stratified meta-analysis results, calculated with linkage disequilibrium score regression (LDSC) (7), were high (word reading $r_g=0.86$, $se=0.16$; nonword reading $r_g=0.88$, $se=0.24$). Regarding test instrument, GWAS meta-analysis results of the most commonly used reading test (Test of Word Reading Efficiency; TOWRE) were found to be highly correlated with GWAS meta-analysis results of all other reading

tests (rg=0.85, se=0.15 for word reading; rg=0.99, se=0.21 for nonword reading). Similar results were obtained when comparing GWAS meta-analysis results of time-restricted reading tests to all other reading tests. Limited heterogeneity of GWAS meta-analysis results was also evident in the Cochran Q statistics (*SI Appendix,* Fig. S3) and LDSC ratios (*Dataset S4*) for all traits except nonword repetition.

We also assessed whether there was genetic heterogeneity related to sex effects, in this case by generating corresponding GWAS data for female- and male-only subsets and estimating the genetic correlations between them. Results of male and female subsets were highly correlated (estimates of genetic correlation are rg=1.04, se=0.17, $p=1.7x10^{-9}$ for word reading; rg=0.97, se=0.19, $p=2.1x10^{-7}$ for nonword reading; rg=1.11, se=0.29, $p=1.0x10^{-4}$ for spelling; rg=1.56, se=0.55, $p=4.2x10^{-3}$ for phoneme awareness; and rg=1.30, se=0.63, p=0.039 for nonword repetition; some estimates exceed 1, because the genetic covariance estimator is not constrained in LDSC, likely representing sampling variation and randomness; *Dataset S4*).

Adolescent Brain Cognitive Development ^{SM} Study (ABCD Study)
Data used in the preparation of this article were obtained from the Adolescent Brain Cognitive Development^{SM} (ABCD) Study (https://abcdstudy.org), held in the NIMH Data Archive (NDA). This is a multisite, longitudinal study designed to recruit more than 10,000 children age 9-10 and follow them over 10 years into early adulthood. A full list of supporters is available at https://abcdstudy.org/federal-partners.html. A listing of participating sites and a complete listing of the study investigators can be found at https://abcdstudy.org/consortium_members/. ABCD consortium investigators designed and implemented the study and/or provided data but did not necessarily participate in the analysis or writing of this report. This manuscript reflects the views of the authors and may not reflect the opinions or views of the NIH or ABCD consortium investigators. The ABCD data used in this report are summarized in this NDA study (https://doi.org/10.15154/1522876).

In the ABCD Study, word reading was assessed using the NIH Toolbox Oral Reading Recognition Test (TORRT), which measures the accuracy of pronouncing single printed words (30). Participants could take as much time as wanted. Age-corrected standard scores were supplied. The Institutional Review Board at the University of California, San Diego approved the study, with a few sites obtaining approval of their local Institutional Review Board. All participants and/or their parents provided informed consent.

Avon Longitudinal Study of Parents and Children (ALSPAC)
ALSPAC (31, 32) is a longitudinal cohort, in which reading- and language-related traits were assessed at multiple specific ages using various tools. Where possible, ages and tools were chosen that optimally matched the data of the other cohorts. The results of the Test of Word Reading Efficiency (TOWRE) (33), assessed at 13 years of age, were used as measures for word and nonword reading. The TOWRE measures the ability to sound out words and nonwords quickly and accurately, by asking participants to read through a list of words for 45 seconds, followed by a list of nonwords for 45 seconds. Spelling was assessed at 7 years of age with the Wechsler Objective Reading Dimensions (WORD) test (34), which contains 6 items assessing the ability to write letters and 44 items assessing spelling. Phoneme awareness was measured at 7 years with the Auditory Analysis Test (35), a phoneme deletion task with 2 practice and 40 test items of increasing difficulty. Participants were asked to repeat each word in full, and then with a phoneme removed. Nonword repetition was assessed using 36 nonwords of 3, 4 and 5 syllables (12 nonwords each) (36). Participants were asked to listen to and then repeat each nonword. Performance IQ was assessed using the Wechsler Intelligence Scale for Children (WISC) (37) at 8 years of age. Please note that the study website contains details of all the data that is available through a fully searchable data dictionary and variable search tool (http://www.bristol.ac.uk/alspac/researchers/our-data/).

Pregnant women resident in Avon, UK with expected dates of delivery 1st April 1991 to 31st December 1992 were invited to take part in the study. The initial number of pregnancies enrolled is 14,541 (for these at least one questionnaire has been returned or a “Children in Focus” clinic had

been attended by 19/07/99). Of these initial pregnancies, there was a total of 14,676 foetuses, resulting in 14,062 live births and 13,988 children who were alive at 1 year of age. When the oldest children were approximately 7 years of age, an attempt was made to bolster the initial sample with eligible cases who had failed to join the study originally. As a result, when considering variables collected from the age of seven onwards (and potentially abstracted from obstetric notes) there are data available for more than the 14,541 pregnancies mentioned above. The number of new pregnancies not in the initial sample (known as Phase I enrolment) that are currently represented on the built files and reflecting enrolment status at the age of 24 years is 913 (456, 262 and 195 recruited during Phases II, III and IV respectively), resulting in an additional 913 children being enrolled. The phases of enrolment are described in more detail in the cohort profile paper and its update. The total sample size for analyses using any data collected after the age of 7 years is therefore 15,454 pregnancies, resulting in 15,589 foetuses. Of these 14,901 were alive at 1 year of age.

Ethical approval for the study was obtained from the ALSPAC Ethics and Law Committee and the Local Research Ethics Committees. Consent for biological samples has been collected in accordance with the Human Tissue Act (2004). Informed consent for the use of data collected via questionnaires and clinics was obtained from participants following the recommendations of the ALSPAC Ethics and Law Committee at the time. We are extremely grateful to all the families who took part in this study, the midwives for their help in recruiting them, and the whole ALSPAC team, which includes interviewers, computer and laboratory technicians, clerical workers, research scientists, volunteers, managers, receptionists and nurses. GWAS data were generated by Sample Logistics and Genotyping Facilities at Wellcome Sanger Institute and LabCorp (Laboratory Corporation of America) using support from 23andMe. The publication is the work of the authors and they will serve as guarantor for the ALSPAC contribution to this article.

The ASTON cohort and Oxford Dyslexia cohort (ASTON and UKDYS cohorts)
In the ASTON and UKDYS cohorts, word reading, spelling and performance IQ was assessed using the British Ability Scale (BAS) (38). The BAS includes an untimed single word reading test. In the spelling test, participants were asked to spell single words dictated in a sentence frame. A matrices test was used to assess performance IQ. Nonword reading was assessed using the CORE (39), lengthened to a 30-item version by Castles and Coltheart (40). This is an untimed nonword reading task, where participants were asked to read aloud 30 nonwords consisting of one and two syllables. Phoneme awareness was assessed using a spoonerism test, where participants were asked to swap the first sounds of two words. Ethical approval was obtained from the Oxfordshire Psychiatric Research Ethics Committee and the Aston University Human Sciences Ethics Committee. All participants and/or their caregivers provided written informed consent.

Brisbane Adolescent Twin Sample (BATS)
In BATS, word and nonword reading and spelling were assessed using the CORE (39), which was lengthened to a 120-word version by Castles and Coltheart (40) to include spelling and to increase the difficulty level for an older sample. Nonword repetition was assessed using the Dollaghan and Campbell nonword repetition task (41). Both tests were administered over the telephone by a trained researcher. The study was approved by the Human Research and Ethics Committee of the QIMR Berghofer research institute; all participants provided informed consent.

Basque Center on Cognition, the Brain and Language cohort (BCBL)
In the BCBL cohort, word and nonword reading was assessed by asking participants to read a list of 192 strings of letters that appeared in the middle of the screen, some of which were real words (96) and some of which were nonwords (96). The words included regular and irregular words. Phoneme awareness was measured using a phoneme deletion task of 24 items. Participants were asked to repeat each word without the first phoneme out loud as fast as possible. Nonword repetition was assessed by asking participants to repeat nonwords of increasing difficulty and length that were presented auditorily through headphones. A total of 24 nonwords were presented to each participant in the same order. For the reading and phoneme awareness tests, participants

had a response time of 3 seconds for each item, and 5 seconds per item in the nonword repetition test. For each test, the accuracy measure was used. Performance IQ was measured with the matrices subtest of the Kaufman Brief intelligence test (K-BIT) (42). The Institutional Review Board of the University of La Laguna approved the study and all participants provided informed consent.

Colorado Learning Disabilities Research Center cohort (CLDRC)

In the CLDRC cohort, word reading was measured with two different tests: the Time-Limited Word Recognition Test (TLWRT) (43, 44) and the Word Recognition subtest of the Peabody Individual Achievement Test (PIAT) (45) word reading test. The TLWRT measures reading accuracy for words where the correct response was initiated within two seconds. The Word Recognition subtest of the PIAT is an untimed word reading test. A composite measure was used, derived in equal weights from the scores on both tests, which has proven to be a more reliable index for word reading than either measure alone (43). Nonword reading was assessed using the TLWRT (44), similarly to the word reading assessment. Spelling was assessed using the Wide Range Achievement Test-Revisited (WRAT-R) (46), where participants were asked to write down words that were dictated. Phoneme awareness was assessed using a classical phoneme deletion task, and nonword repetition was measured using a classical nonword repetition task. Performance IQ was measured with the WISC. The study was approved by the Institutional Review Board of the University of Colorado; all participants provided informed consent.

Early Language in Victoria Study (ELVS)

ELVS is a longitudinal cohort, in which reading- and language-related traits were assessed at specific ages using various tools. Word reading and spelling was assessed using the WRAT-4 (46) at 11 years of age. In the word reading subtest, participants are asked to pronounce 55 words that are presented without context. In the spelling subtest, participants are asked to write down their name, 13 letters and 42 dictated words of increasing difficulty. Participants get 10 seconds for reading each word, 5 seconds for writing letters and 15 seconds for spelling words. Nonword reading was assessed at 7 years of age using the Castle and Coltheart's reading test (40). Phoneme awareness was measured at 5 years of age using the blending words subtest of the Comprehensive Test of Phonological Processing (CTOPP) (47), where participants have to put individual sounds together to form words. Nonword reading was measured with the Children's Test of Nonword Repetition (CNRep) (48) at 5 years of age. In this task, children are asked to repeat 40 nonwords and their accuracy is scored. Performance IQ was measured using the Wechsler Adult Intelligence Scale (WAIS) (49) at 11 years of age. Standard scores were available for all measures except nonword reading. The study was approved by the Royal Children's Hospital Human Research Ethics Committee.

Familial Influences on Literacy Abilities project (FIOLA)

In FIOLA, word reading was assessed using the One-Minute-Test, or Eén-Minuut-Test in Dutch (EMT) (50), and nonword reading was assessed using the Klepel (51). Participants were asked to correctly read as many (non)words as possible within one minute (for word reading) or two minutes (for nonword reading). The original test versions consist of a list of 116 (non)words of increasing difficulty. To avoid a ceiling effect in adults, the lists were extended by adding the last column of its parallel test, resulting in a list of 145 (pseudo)words. Phoneme awareness was measured using a phoneme-deletion test. On each test item a phoneme (always a consonant) had to be deleted from a pseudoword, resulting in another pseudoword. The test consisted of two parts. The first part comprised four monosyllabic and four disyllabic pseudowords. The second part consisted of four disyllabic pseudowords, with the phoneme that had to be deleted occurring twice. Both parts of the test started with two items (with feedback) for practice. For measuring reaction times, the timer started running after the audio file finished, until right after the participant finished pronouncing the answer. Ethical approval for this study was provided by the University of Amsterdam's Ethics Committee. Written informed consent was obtained from parents. We are grateful to the NEMO Science Museum.

Generation R (GenR)
GenR is conducted by the Erasmus Medical Center in close collaboration with School of Law and Faculty of Social Sciences of the Erasmus University Rotterdam, the Municipal Health Service Rotterdam area, the Rotterdam Homecare Foundation, and the Stichting Trombosedienst & Artsenlaboratorium Rijnmond (STAR[1]MDC), Rotterdam. In GenR, nonword repetition was measured using the Shortened Nonword Repetition Task (NWR-S) (52), a shortened version of test developed by Rispens and Baker (53), in which 22 out of the original 40 nonwords were included. The percentage of nonwords repeated correctly was used as outcome. The study was approved by the Medical Ethical Committee of the Erasmus Medical Center in Rotterdam. All participants and/or their parents provided written informed consent.

Genes, Reading, and Dyslexia study (GRaD)
In GRaD, word and nonword reading was measured with the TOWRE (33). Spelling was assessed with the Woodcock Johnson-III (WJ-III) (54), in which increasingly difficult words are dictated to the participant in the context of a sentence, and the participant is asked to write down the words. Phoneme awareness was measured using the CTOPP (47) elision task, during which the participants are asked to repeat a spoken word while omitting a target sound. The GRaD study was approved by the Yale Human Investigation Committee and all the review boards of participating data collection sites.

Iowa Study
In the Iowa study, word and nonword reading was measured with the Woodcock Reading Mastery Tests-Revisited (WRMT-R) (55) using the Word Identification and Word Attack subtests, respectively. In these tests, participants are asked to read aloud isolated words or nonwords from a list with increased difficulty. Spelling was assessed using the Test of Written Spelling-2 (TWS2) (56), during which participants are asked to write down dictated words with increasing difficulty. Phoneme awareness was measured using the Catts elision task (57), during which participants are asked to repeat a word while omitting a target sound. Nonword repetition was measured using the Dollaghan and Campbell nonword repetition task (41). Performance IQ was measured with the WISC (37). The study was approved by the Institutional Review Board of the University of Iowa IRB-01 (#200511767). All subjects in the Iowa cohort were minors who assented to participation.

NeuroDys
NeuroDys is a cohort collected in seven different European countries: Austria, Germany, Switzerland, Finland, France, Hungary and the Netherlands. For all traits, language-specific tests were used. Word reading and nonword reading was assessed by presenting language-specific lists of words or nonwords, while participants were asked to read as quickly as possible without making mistakes. Spelling was assessed by asking participants to spell single words dictated in sentence frames. Phoneme awareness was assessed using a phoneme deletion task. See (58, 59) for details. Performance IQ was measured using the Block Design subtest of the WISC (37). Ethical approval was granted by the Research Ethics Committee of the NHS (14/NS/1022), the Kantonale Ethikkomission Zürich, the Ethics Committee of the University of Salzburg, the Ethics Committee of the Hospital District of Central Finland and the Ethics Committee of the Department of Child and Adolescent Psychiatry, Psychosomatic, and Psychotherapy of the Philipps University in Marburg. Informed written consent was given by caregivers.
NeuroDys project partners include Catherine Billard, Caroline Bogliotti, Vanessa Bongiovanni, Laure Bricout, Camille Chabernaud, Yves Chaix, Isabelle Comte-Gervais, Florence Delteil-Pinton, Fabien Fauchereau, Florence George, Christophe-Loïc Gérard, Ferenc Honbolygó, Guillaume Huguet, Stéphanie Iannuzzi, Marie Lageat, Marie-France Leheuzey, Marie-Thérèse Lenormand, Marion Liébert, Emilie Longeras, Emilie Racaud, Isabelle Soares-Boucaud, Sylviane Valdois, Nadège Villiermet, and Johannes Ziegler.

Netherlands Twin Register (NTR)
The NTR is a longitudinal cohort, in which reading- and language-related traits were assessed at multiple specific ages. Where possible, ages were chosen to optimally match the data of the other cohorts. Word reading was measured using the EMT (50) and three-minutes test (Drie-Minuten

Test, DMT) (60) at 9 years of age. During the DMT, participants were asked to correctly read as many words as possible within three minutes. Spelling data of children in grade 3 (comparable to grade 1 in most countries; age 6 to 7 years) were obtained from Cito's pupil monitoring system. Active spelling was measured by asking children to write down specific words dictated within sentence context. Passive spelling was measured with multiple choice questions on the spelling of a highlighted word in a sentence. Performance IQ was assessed between 6 and 18 years of age with the WISC (37), WAIS (49) or Revisie Amsterdamse Kinder Intelligentietest (RAKIT) (61). The study was approved by the Central Ethics Committee on Research Involving Human Subjects of the VU University Medical Centre, Amsterdam, an Institutional Review Board certified by the U.S. Office of Human Research Protections (IRB number IRB00002991 under Federal-wide Assurance-FWA00017598; IRB/institute codes, NTR 03-180). Informed consent was obtained from all parents of twins.

Pediatric Imaging, Neurocognition, and Genetics cohort (PING)
PING data are disseminated by the PING Coordinating Center at the Center for Human Development, University of California, San Diego. In the PING cohort, word reading was assessed using the WRAT-4 (46). During this task, participants were asked to read aloud 55 words from a list. The human research protections programs and institutional review boards at the universities participating in the PING project approved all experimental and consenting procedures. Written parental informed consent was obtained for all PING subjects below the age of 18, and child assent was also obtained for all participants between the ages of 7 and 17. Written informed consent was obtained directly from all participants aged 18 years or older. The dataset analysed was accessed via dbGaP number 19848 and 12043 (Neurodevelopmental Genomics: Trajectories of Complex Phenotypes (phs000607.v3.p2)).

The Philadelphia Neurodevelopmental Cohort (PNC)
All subjects were recruited through the Center for Applied Genomics at The Children's Hospital in Philadelphia. Reading performance was assessed as part of the cognitive test battery and collected by Gur et al. (62). In the PNC, word reading was assessed using the WRAT-4 (46). Participants were asked to read aloud 55 words from a list. The institutional review boards of the University of Pennsylvania and the Children's Hospital of Philadelphia approved all study procedures. All adult participants provided informed consent; for participants under the age of 18 years, assent and parental consent were obtained.

The Raine Study
In the Raine Study, spelling was measured at 10 years of age using the Western Australian Literacy and Numeracy Assessment (WALNA). This spelling test consists of two parts: in the first part participants were asked to correct the spelling of 10 highlighted words from a written text that was also read aloud, and in the second part participants were asked to write down 14 words that were missing from written text but present when that text was read aloud. Performance IQ was assessed using Raven's Coloured Progressive Matrices (CPM) (63). See (64) for more details. The study was approved by the Human Research Ethics Committee of King Edward Memorial Hospital, Princess Margaret Hospital, the University of Western Australia, and the Health Department of Western Australia. Consent was provided by all participants at each follow-up within the Raine Study.

SLI consortium (SLIC)
In the SLIC cohort, word reading and spelling was measured with the WORD test (34). The word reading test consists of three parts: the first part has 4 items assessing the match between letters and beginning and end sounds, the second part has 3 items matching pictures to words, and the third part is a word reading test of 48 items increasing in difficulty. The spelling test contains 6 items to assess the ability to write letters and 44 items assessing spelling. Nonword repetition was assessed by asking participants to repeat 28 nonwords that were delivered orally or by tape. Performance IQ was assessed using the WISC (37).

Ethical permission for each collection was granted by local ethics committees. Guys Hospital Research Ethics Committee approved the collection of families from the Newcomen Centre to identify families from the South East of England with specific language disorder (Ref No. 96/7/11). Cambridge Local Research Ethics Committee approved the CLASP project “Genome Search for susceptibility loci to language disorders” (Ref No. LREC96/212). Ethical approval for the Manchester Language Study was given by the University of Manchester Committee on the Ethics of Research on Human Beings (Ref No. 03061). The Lothian Research Ethics Committee approved the project “Genetics of specific language impairment in children in Scotland” for the use of the Edinburgh samples (Ref. No. LREC/1999/6/20). All participants provided informed consent.

SLI Consortium members are as follows: Wellcome Trust Centre for Human Genetics, Oxford: D. F. Newbury, N. H. Simpson, F. Ceroni, A. P. Monaco; Max Planck Institute for Psycholinguistics, Nijmegen: S. E. Fisher, C. Francks; Newcomen Centre, Evelina Children’s Hospital, St Thomas’ Hospital, London: G. Baird, V. Slonims; Child and Adolescent Psychiatry Department and Medical Research Council Centre for Social, Developmental, and Genetic Psychiatry, Institute of Psychiatry, London: P. F. Bolton; Medical Research Council Centre for Social, Developmental, and Genetic Psychiatry Institute of Psychiatry, London: E. Simonoff; Salvesen Mindroom Centre, Child Life & Health, School of Clinical Sciences, University of Edinburgh: A. O’Hare; Cell Biology & Genetics Research Centre, St. George’s University of London: J. Nasir; Queen’s Medical Research Institute, University of Edinburgh: J. Seckl; Department of Speech and Language Therapy, Royal Hospital for Sick Children, Edinburgh: H. Cowie; Speech and Hearing Sciences, Queen Margaret University: A. Clark, J. Watson; Department of Educational and Professional Studies, University of Strathclyde: W. Cohen; Department of Child Health, the University of Aberdeen: A. Everitt, E. R. Hennessy, D. Shaw, P. J. Helms; Audiology and Deafness, School of Psychological Sciences, University of Manchester: Z. Simkin, G. Conti-Ramsden; Department of Experimental Psychology, University of Oxford: D. V. M. Bishop; Biostatistics Department, Institute of Psychiatry, London: A. Pickles.

Saguenay Youth Study (SYS)

In SYS, spelling was assessed using the Woodcock Johnson spelling test (54), during which participants were asked to spell and write down a list of 45 words. The number of correct answers was used as a measure for spelling. Performance IQ was measured using the WISC-III (37). The study was approved by the Research Ethics Boards of the Chicoutimi Hospital in Chicoutimi, Quebec, Canada and the Hospital for Sick Children in Toronto, Ontario, Canada. All participants provided informed consent.

Twins Early Development Study (TEDS)

TEDS is a longitudinal cohort, in which reading- and language-related traits have been assessed at multiple specific ages using various tools. Where possible, ages and tools were chosen that optimally matched the data of the other cohorts. Word reading and nonword reading were assessed using the TOWRE (33) at 12 years of age. Spelling was assessed using the Key stage one at 7 years of age; these data were derived from the National Pupil database. During this test, children are asked to write down 20 words that are missing from a sentence. Performance IQ was measured using the WISC (37) at 12 years of age. Ethical approval for TEDS was provided by the King’s College London Ethics Committee (Ref No. PNM/09/10–104). Participants have provided informed consent at each wave of assessment.

Toronto cohort

In the Toronto cohort, word reading and nonword reading were assessed using the TOWRE (33). Phoneme awareness was measured using the CTOPP (47) phoneme awareness composite score, a standard score based on the elision, blending words and sound matching subtests. Nonword repetition was assessed using the CTOPP nonword repetition task, during which participants are asked to repeat 18 nonwords. Performance IQ was measured using the WISC (37) III and IV. Procedural approval was given by the Hospital for Sick Children and University Health Network Research Ethics Boards. Verbal assent and/or written consent was obtained from all children and parents.

York cohort

York is a longitudinal cohort, in which reading- and language-related traits were assessed at multiple specific ages using various tools. Where possible, ages and tools were chosen that optimally matched the data of the other cohorts. Word and nonword reading were assessed using the TOWRE (33). Spelling was assessed using the Wechsler Individual Achievement Test (WIAT) (65), during which letters, sounds and words are dictated in a sentence framework. Phoneme awareness was measured using a phoneme deletion task. Nonword repetition was assessed using the CNRep (48). Performance IQ was measured using the WISC (37). Ethical approval for the study was obtained from the Research Ethics Committee of the NHS (Yorkshire and the Humber – Humber Bridge) and the Ethics Committee of the Department of Psychology of the University of York. Parents provided written informed consent.

Supplemental Figures

Word reading

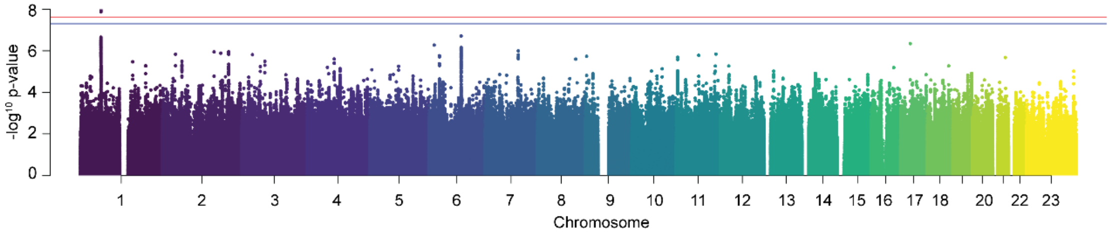

Nonword reading

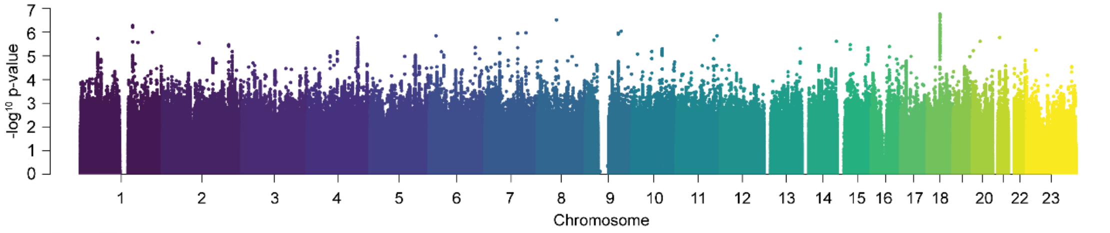

Spelling

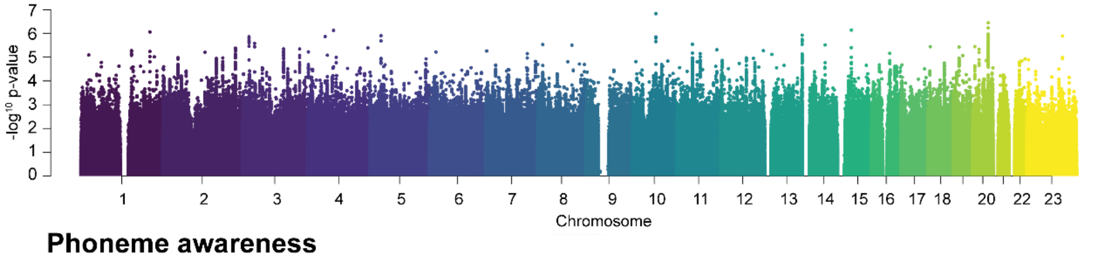

Phoneme awareness

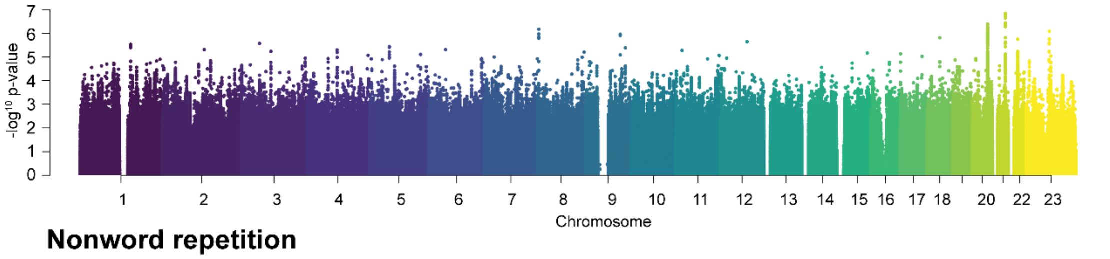

Nonword repetition

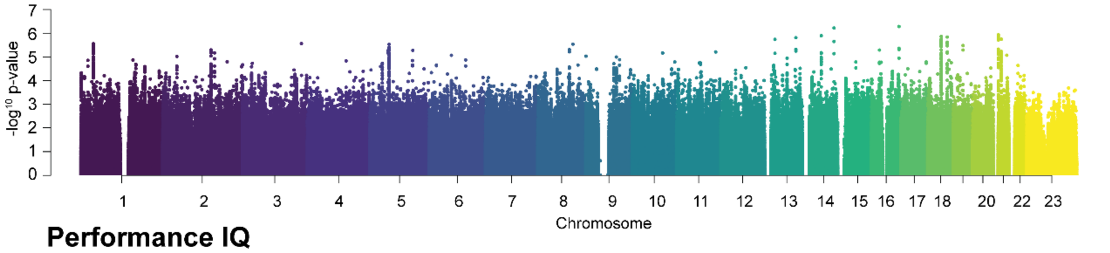

Performance IQ

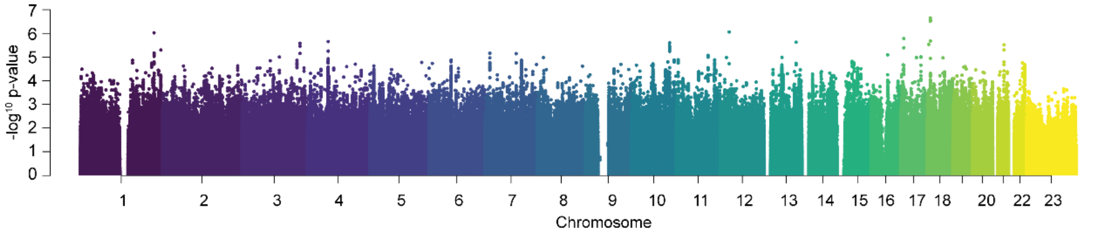

Figure S1. Manhattan plots of the results of the GenLang GWAS meta-analyses. The x axis shows chromosomal position (hg19) and the y axis represents -log(two-sided P values) for association of variants with the traits. The horizontal red line represents the threshold for genome-wide significance after correction for 2.15 independent GenLang traits ($p<2.33x10^{-8}$). The horizontal blue line represents the standard threshold for genome wide significance ($p<5x10^{-8}$).

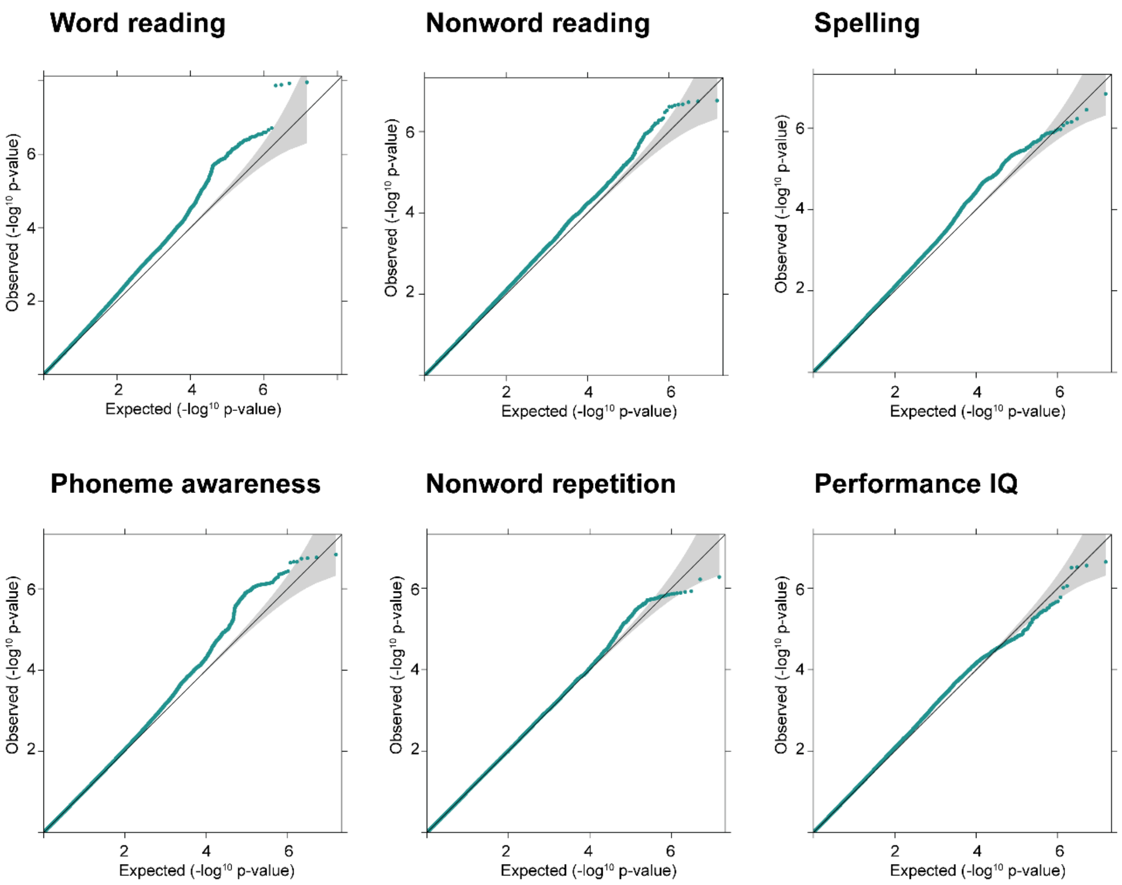

Figure S2. Quantile-quantile plots of each of the GenLang meta-analyses. The gray-shaded areas in the plots represent the 95% confidence intervals under the null hypothesis.

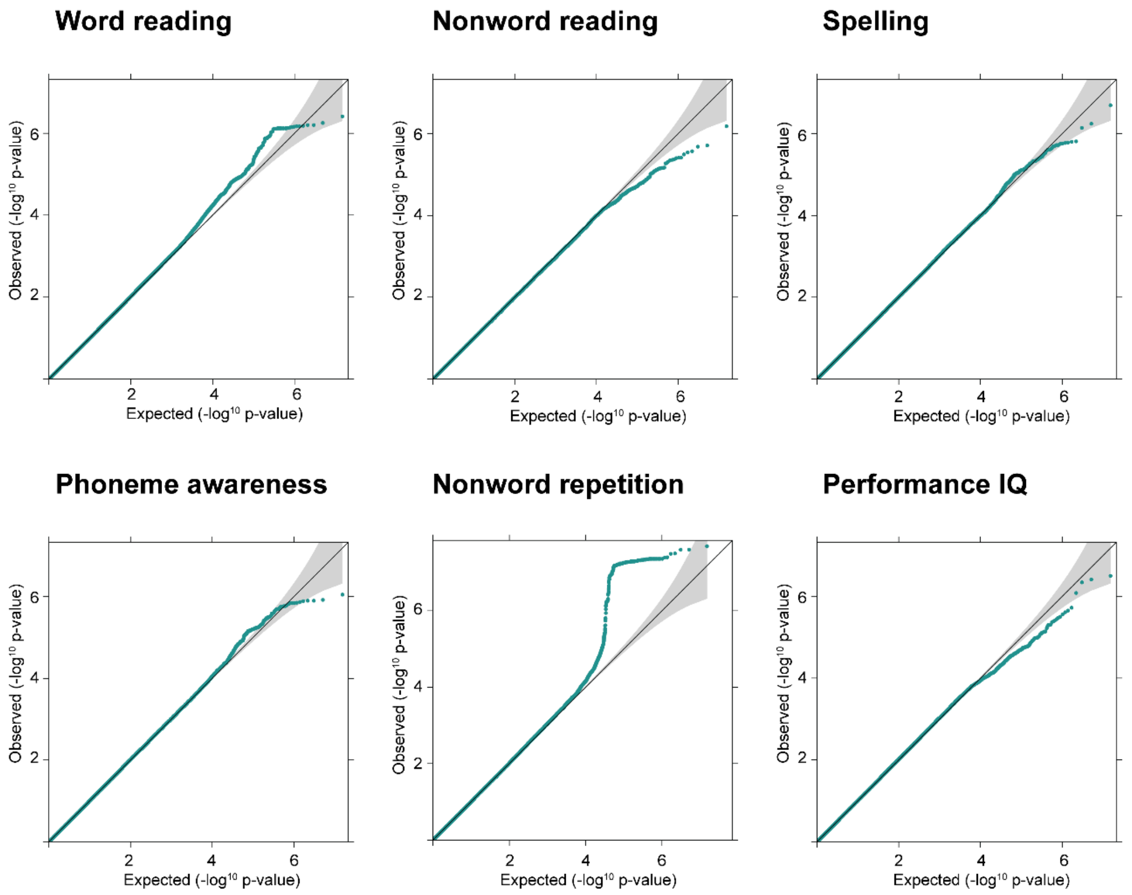

Figure S3. Quantile-quantile plots of the Cochran's Q-test p-values for each of the GenLang meta-analyses. The gray-shaded areas in the plots represent the 95% confidence intervals under the null hypothesis. For nonword repetition, a random-effects meta-analysis was performed because of the high heterogeneity.

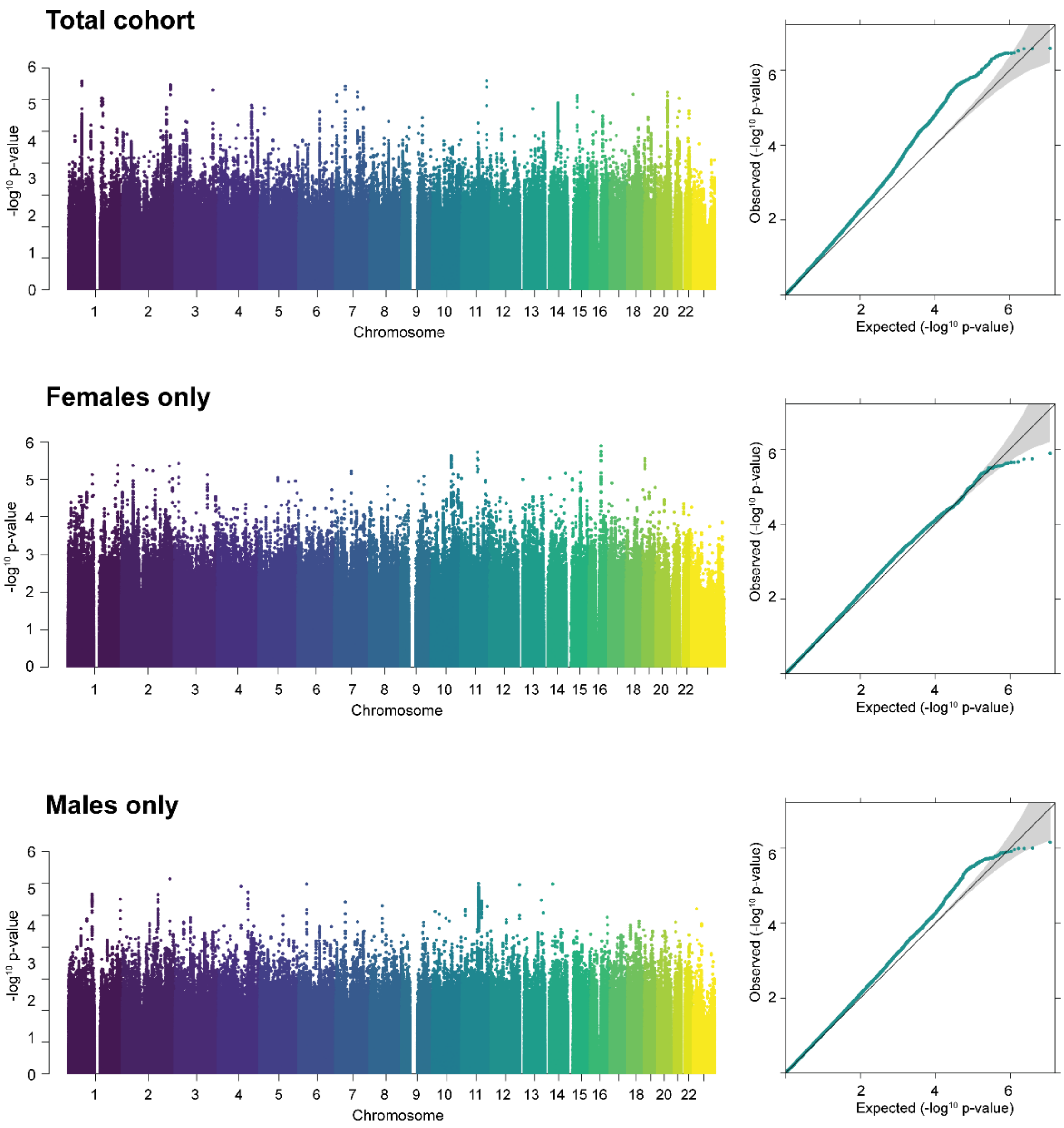

Figure S4. Manhattan and quantile-quantile plots of the multivariate GWAS results. The multivariate GWAS analysis was performed using MTAG (2), combining the univariate GWAS meta-analysis results of the four most highly correlated traits: word reading, nonword reading, spelling and phoneme awareness. Left: Manhattan plot of the p-values of the MTAG multivariate GWAS analysis. The y axis represents -log(two-sided P values) for association of variants with the traits. Right: Quantile-quantile plots of the p-values of the MTAG multivariate GWAS analysis. The gray-shaded areas represent the 95% confidence intervals under the null hypothesis. The plots are shown of the MTAG results for the total cohort and for female-only and male-only analyses.

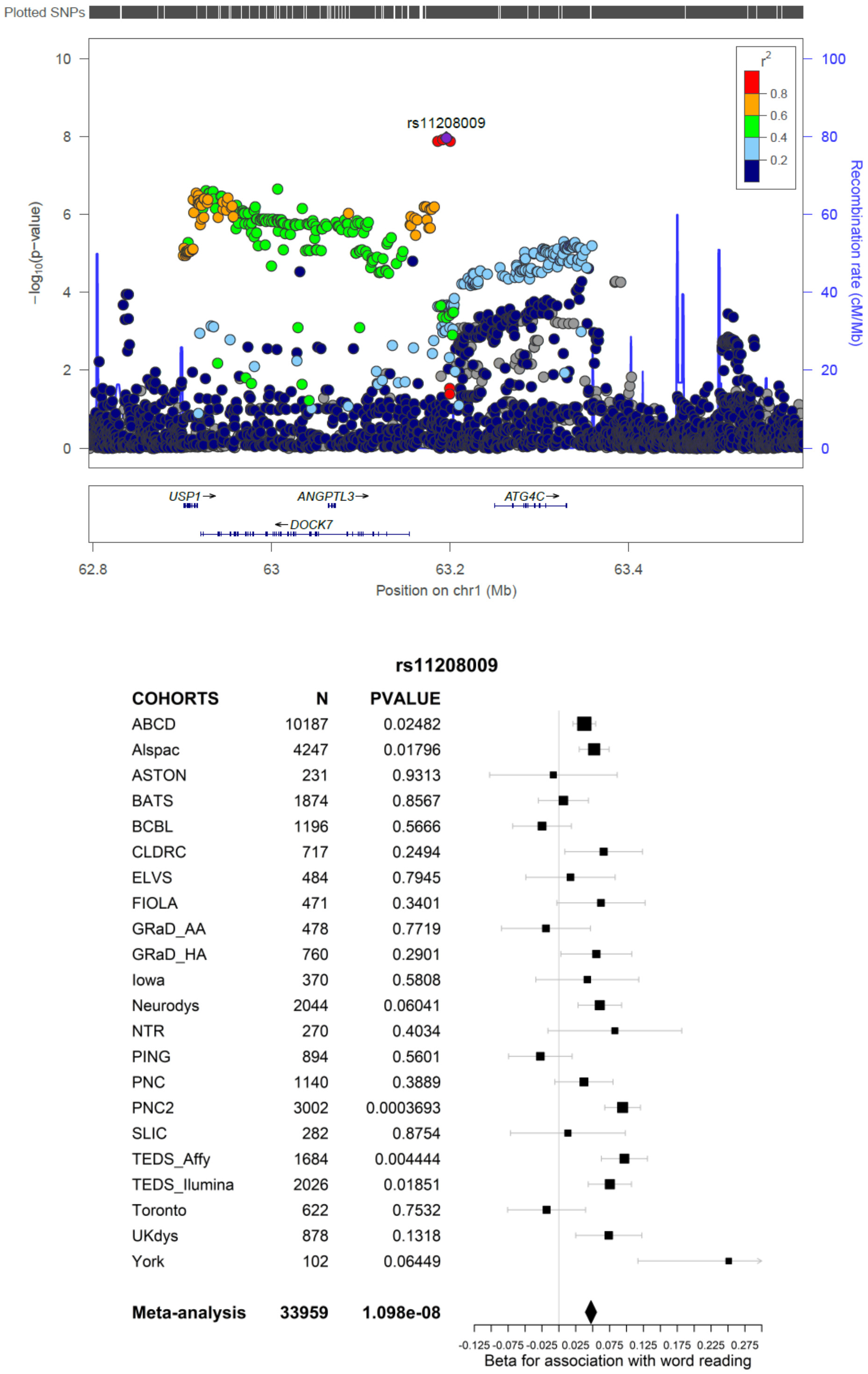

Figure S5. Locuszoom plot and Forestplot of the genome-wide significant locus associated with word reading on chromosome 1. Top: Locuszoom plot. Colours represent linkage disequilibrium with rs11208009 based on the 1000 Genomes project reference data. Bottom: Forest plot of the association results for rs11208009 in each of the GenLang cohorts.

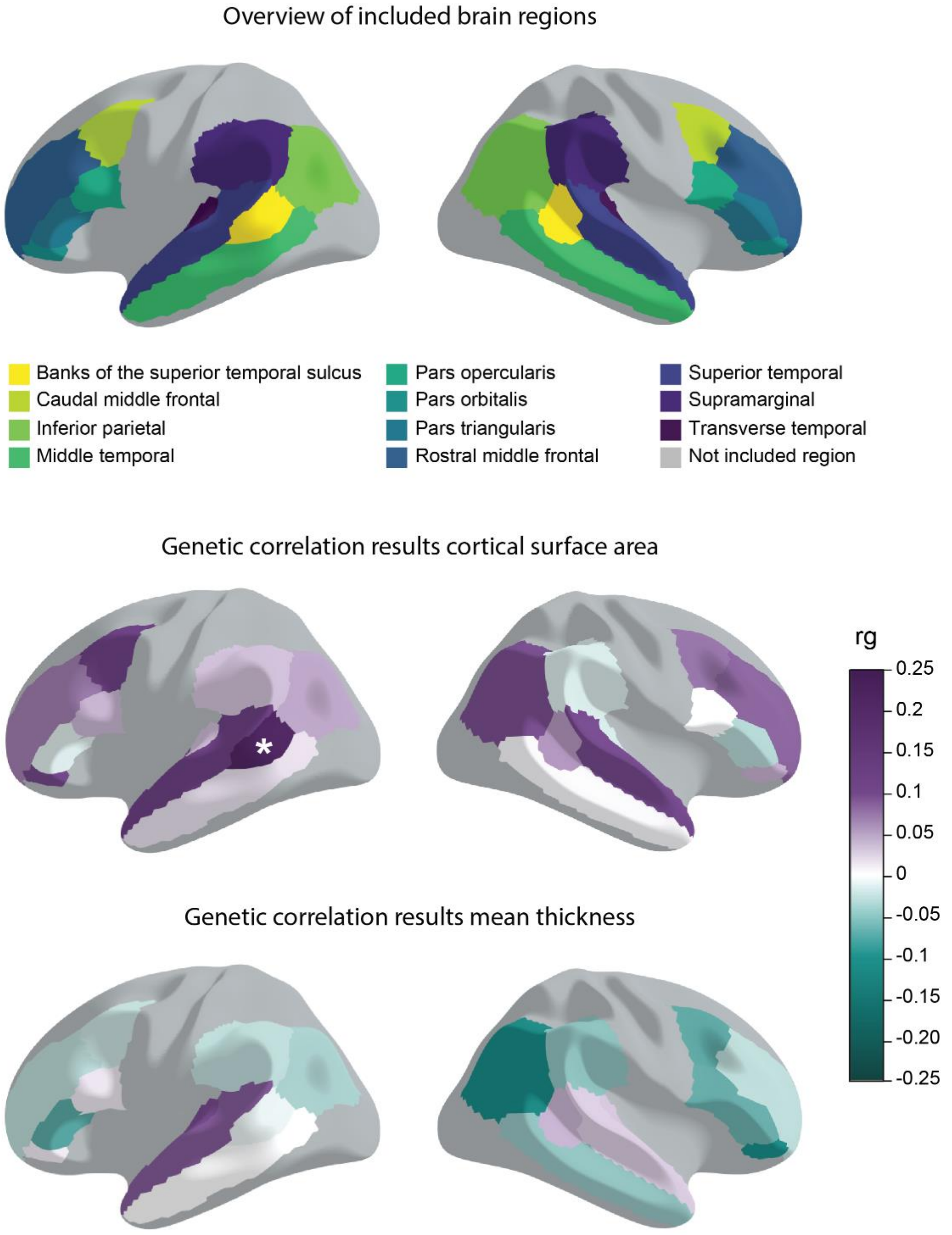

Figure S6. Overview of surface-based morphometric (SBM) neuroanatomical traits, assessed via brain imaging, and their genetic correlations with the multivariate GenLang results. Top: Overview of the cortical areas included in the genetic correlation analysis with the multivariate GenLang results. The 11 regions were selected based on a literature review encompassing brain regions and white matter tracts with known links to aspects of reading and language (see Methods). For each of the 11 regions, GWAS summary statistics of cortical surface area and mean thickness from the UK Biobank were obtained for the left and right hemisphere. Genetic correlations (rg) with the multivariate GenLang results were estimated with LD score regression. Results are shown for cortical surface (middle) and mean thickness (bottom). Purple to green colours represent genetic correlations (rg) with the multivariate GenLang results. Grey areas are not included in the genetic correlation analyses. *$p<2.01x10^{-3}$ ($p<0.05$ after correction for 24.85 independent brain imaging traits). Results can also be found in *Dataset S12*.

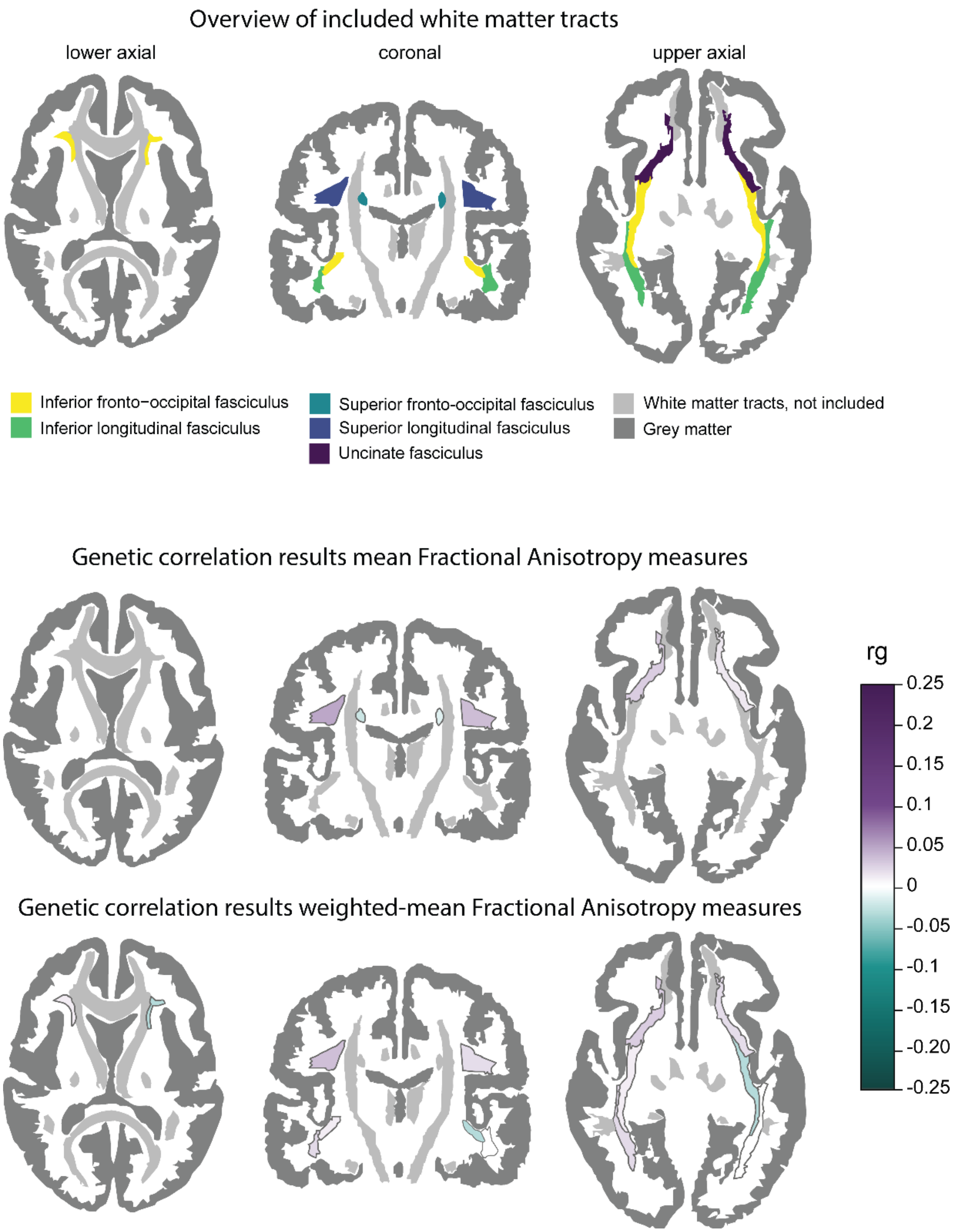

Figure S7. Overview of diffusion tensor imaging (DTI) traits and their genetic correlation with the multivariate GenLang results. Top: Overview of the DTI traits included in the genetic correlation analysis with the multivariate GenLang results. Included are GWAS summary statistics of 14 DTI traits for five different white matter tracts from the UK Biobank, selected based on a literature review encompassing brain regions and white matter tracts with known links to aspects of reading and language (see Methods). Middle and bottom: overview of the genetic correlations (rg) between the multivariate GenLang GWAS meta-analysis results and the DTI traits, estimated with LD score regression. Separate plots are made for the mean Fractional Anisotropy (FA) measures (middle) and the weighted-mean FA measures (bottom). Results can also be found in *Dataset S12*.

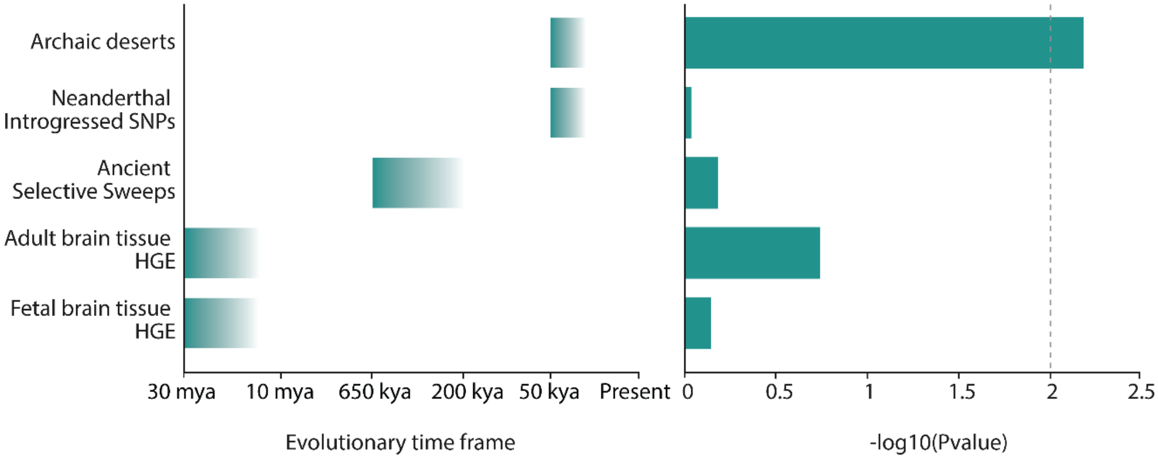

Figure S8. LDSC heritability partitioning of the multivariate GenLang GWAS identifies significant enrichment in archaic deserts. Five annotations were studied, reflecting different aspects of human evolution spanning diverse periods from 30 million to 50,000 years ago. Left: Schematic overview of the approximate time frames captured by the five annotations, as a rough guide to the relative periods involved; note that precise boundaries for these are not defined. Mya: million years ago; kya: thousand years ago; HGE: human gained enhancers. Right: The -log-10 p-values of the heritability partitioning analysis of the multivariate GenLang results. The dashed line shows the p-value threshold for significant enrichment after Bonferroni-correction for testing 5 annotations. Results are also available in *Dataset S14*.

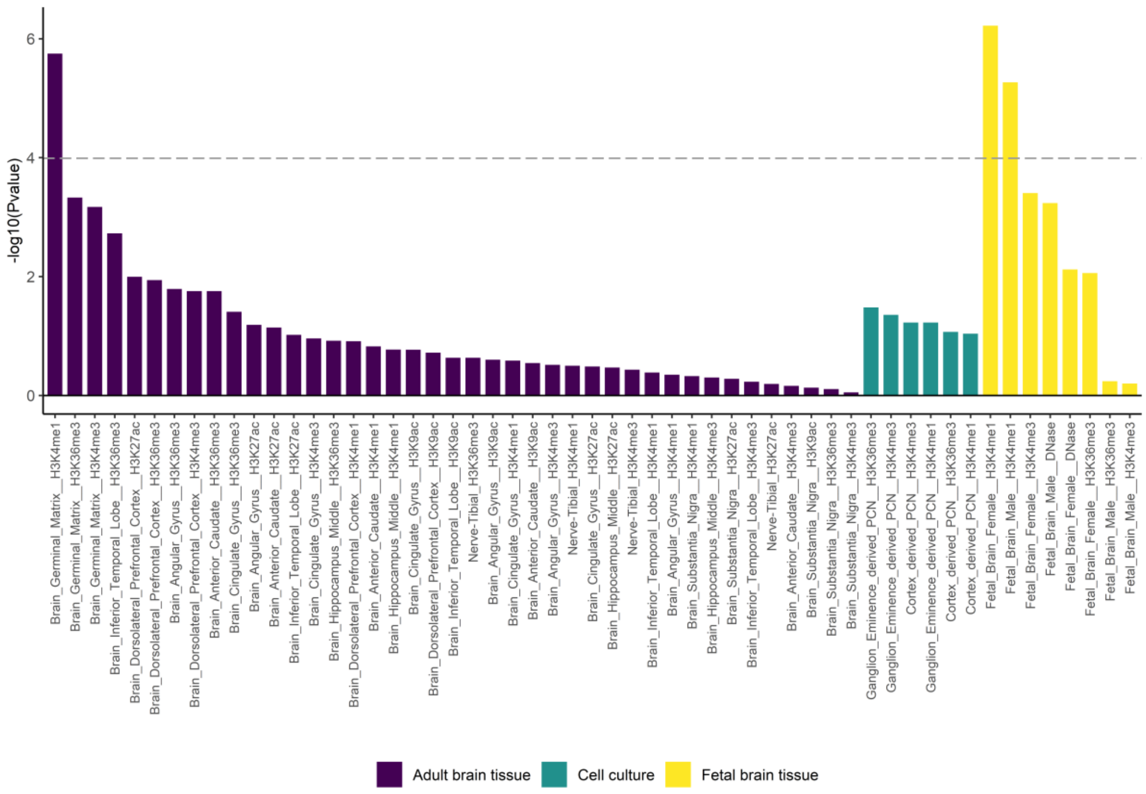

Figure S9. SNP-based heritability of the multivariate GenLang GWAS is significantly enriched in brain enhancers. 489 annotations of tissue-specific chromatin signatures were used to analyse the multivariate GWAS results with LDSC heritability partitioning. Only brain annotations are shown; full results are available in *Dataset S14*. PCN: primary cultured neurospheres. The graph shows log-10 p-values on the y-axis and brain region-specific chromatin mark on the x-axis. The dashed line shows the p-value threshold for significant enrichment after Bonferroni correction for testing 489 annotations.

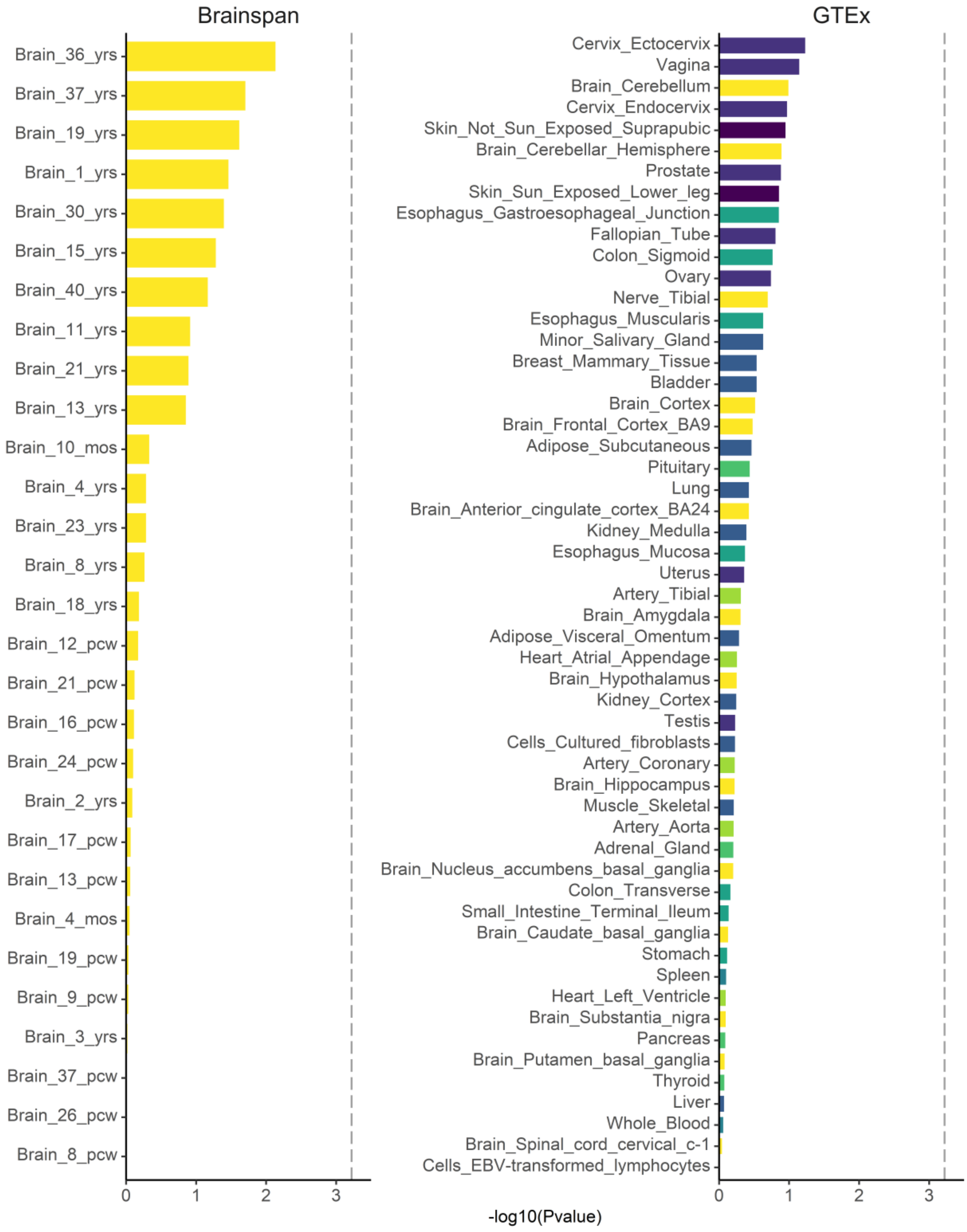

Figure S10. MAGMA gene property analysis of the multivariate GenLang results using two RNA-sequencing datasets of human tissue. Brainspan data include human brain tissue at different ages during development, and GTEx V8 data include a wide range of adult human tissues. The graph shows -log-10 p-values on the y-axis and cell types on the x-axis. The dashed line shows the p-value threshold for significant enrichment after Bonferroni correction for testing 83 tissues.

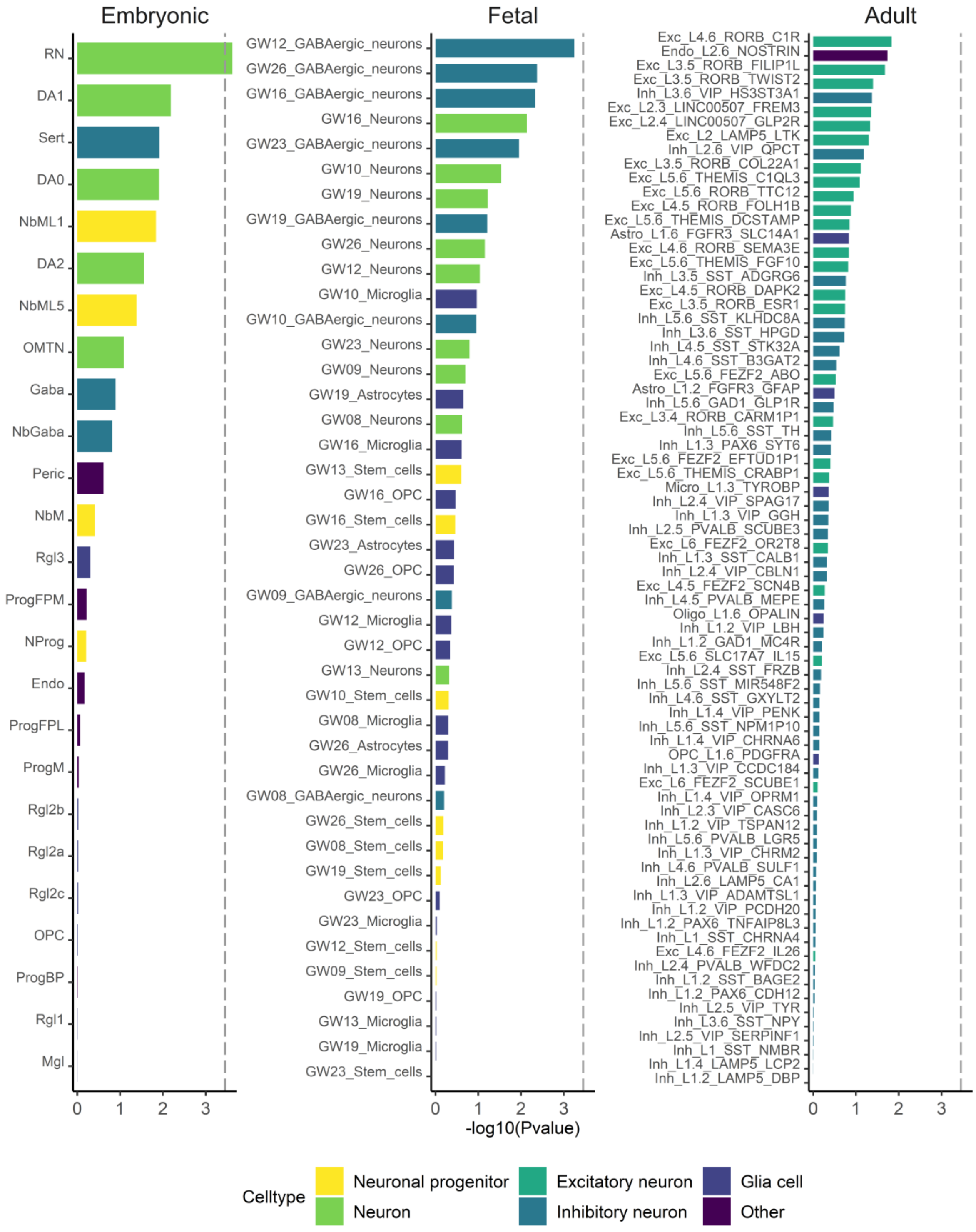

Figure S11. Gene property analysis with MAGMA identifies a relation between the multivariate GenLang results and embryonic red nucleus neurons. MAGMA gene property analysis using three single-cell RNA-sequencing datasets of human brain tissue, representing embryonic midbrain and fetal and adult cortex. The graph shows -log-10 p-values on the y-axis and cell types on the x-axis. The dashed line shows the p-value threshold for significant enrichment after Bonferroni correction for testing 142 cell types.

SI References

1. B.K. Bulik-Sullivan, *et al.* Ld score regression distinguishes confounding from polygenicity in genome-wide association studies. *Nature genetics* 47(3):291-295 (2015).
2. P. Turley, *et al.* Multi-trait analysis of genome-wide association summary statistics using mtag. *Nature genetics* 50(2):229-237 (2018).
3. C.A. de Leeuw, J.M. Mooij, T. Heskes, & D. Posthuma Magma: Generalized gene-set analysis of gwas data. *PLoS computational biology* 11(4):e1004219 (2015).
4. T.W. Winkler, *et al.* Quality control and conduct of genome-wide association meta-analyses. *Nature protocols* 9(5):1192-1212 (2014).
5. C.J. Willer, Y. Li, & G.R. Abecasis Metal: Fast and efficient meta-analysis of genomewide association scans. *Bioinformatics* 26(17):2190-2191 (2010).
6. J. Zheng, *et al.* Phenospd: An integrated toolkit for phenotypic correlation estimation and multiple testing correction using gwas summary statistics. *Gigascience* 7(8) (2018).
7. B. Bulik-Sullivan, *et al.* An atlas of genetic correlations across human diseases and traits. *Nature genetics* 47(11):1236-1241 (2015).
8. A.D. Grotzinger, *et al.* Genomic structural equation modelling provides insights into the multivariate genetic architecture of complex traits. *Nat Hum Behav* 3(5):513-525 (2019).
9. J.J. Lee, *et al.* Gene discovery and polygenic prediction from a genome-wide association study of educational attainment in 1.1 million individuals. *Nature genetics* 50(8):1112-1121 (2018).
10. P.A. Demange, *et al.* Investigating the genetic architecture of noncognitive skills using gwas-by-subtraction. *Nature genetics* 53(1):35-44 (2021).
11. V.M. Rajagopal, *et al.* Genome-wide association study of school grades identifies a genetic overlap between language ability, psychopathology and creativity. *bioRxiv*:2020.2005.2009.075226 (2020).
12. S.M. Smith, *et al.* An expanded set of genome-wide association studies of brain imaging phenotypes in uk biobank. *Nature neuroscience* 24(5):737-745 (2021).
13. D. Roehrich-Gascon, S.L. Small, & P. Tremblay Structural correlates of spoken language abilities: A surface-based region-of interest morphometry study. *Brain and language* 149:46-54 (2015).
14. M.V. Perdue, J. Mednick, K.R. Pugh, & N. Landi Gray matter structure is associated with reading skill in typically developing young readers. *Cereb Cortex* 30(10):5449-5459 (2020).
15. F.M. Richardson & C.J. Price Structural mri studies of language function in the undamaged brain. *Brain Struct Funct* 213(6):511-523 (2009).
16. C.J. Price The anatomy of language: A review of 100 fmri studies published in 2009. *Ann N Y Acad Sci* 1191:62-88 (2010).
17. S.J. Forkel & M. Catani (2019) Diffusion imaging methods in language sciences. *The oxford handbook of neurolinguistics* eds de Zubicaray GI & Schiller NO (Oxford University Press, Oxford).

18. J. Zheng, *et al.* Ld hub: A centralized database and web interface to perform ld score regression that maximizes the potential of summary level gwas data for snp heritability and genetic correlation analysis. *Bioinformatics* 33(2):272-279 (2017).
19. W. van Rheenen, W.J. Peyrot, A.J. Schork, S.H. Lee, & N.R. Wray Genetic correlations of polygenic disease traits: From theory to practice. *Nature Reviews Genetics* 20(10):567-581 (2019).
20. K. Watanabe, E. Taskesen, A. van Bochoven, & D. Posthuma Functional mapping and annotation of genetic associations with fuma. *Nature communications* 8(1):1826 (2017).
21. K. Watanabe, M. Umicevic Mirkov, C.A. de Leeuw, M.P. van den Heuvel, & D. Posthuma Genetic mapping of cell type specificity for complex traits. *Nature communications* 10(1):3222 (2019).
22. H.K. Finucane, *et al.* Partitioning heritability by functional annotation using genome-wide association summary statistics. *Nature genetics* 47(11):1228-1235 (2015).
23. H.K. Finucane, *et al.* Heritability enrichment of specifically expressed genes identifies disease-relevant tissues and cell types. *Nature genetics* 50(4):621-629 (2018).
24. A.K. Tilot, *et al.* The evolutionary history of common genetic variants influencing human cortical surface area. *Cereb Cortex* 31(4):1873-1887 (2021).
25. M.W. Vermunt, *et al.* Epigenomic annotation of gene regulatory alterations during evolution of the primate brain. *Nature neuroscience* 19(3):494-503 (2016).
26. S.K. Reilly, *et al.* Evolutionary genomics. Evolutionary changes in promoter and enhancer activity during human corticogenesis. *Science* 347(6226):1155-1159 (2015).
27. S. Peyregne, M.J. Boyle, M. Dannemann, & K. Prufer Detecting ancient positive selection in humans using extended lineage sorting. *Genome research* 27(9):1563-1572 (2017).
28. B. Vernot & J.M. Akey Resurrecting surviving neandertal lineages from modern human genomes. *Science* 343(6174):1017-1021 (2014).
29. B. Vernot, *et al.* Excavating neandertal and denisovan DNA from the genomes of melanesian individuals. *Science* 352(6282):235-239 (2016).
30. R.C. Gershon, *et al.* Iv. Nih toolbox cognition battery (cb): Measuring language (vocabulary comprehension and reading decoding). *Monographs of the Society for Research in Child Development* 78(4):49-69 (2013).
31. A. Boyd, *et al.* Cohort profile: The 'children of the 90s'--the index offspring of the avon longitudinal study of parents and children. *Int J Epidemiol* 42(1):111-127 (2013).
32. A. Fraser, *et al.* Cohort profile: The avon longitudinal study of parents and children: Alspac mothers cohort. *Int J Epidemiol* 42(1):97-110 (2013).
33. J.K. Torgesen, C.A. Rashotte, & R.K. Wagner (1999) *Towre: Test of word reading efficiency* (Pro-ed Austin, TX).
34. D. Wechsler (1993) *Wechsler objective reading dimensions* (The Psychological Corporation, London).
35. J. Rosner & D.P. Simon The auditory analysis test: An initial report. *Journal of Learning disabilities* 4(7):384-392 (1971).

36. S.E. Gathercole, C.S. Willis, A.D. Baddeley, & H. Emslie The children's test of nonword repetition: A test of phonological working memory. *Memory* 2(2):103-127 (1994).
37. D. Wechsler & H. Kodama (1949) *Wechsler intelligence scale for children* (Psychological corporation New York).
38. C.D. Elliott (1979) *British ability scales* (nfer-nelson).
39. T.C. Bates, *et al.* Behaviour genetic analyses of reading and spelling: A component processes approach. *Australian Journal of Psychology* 56(2):115-126 (2004).
40. A. Castles & M. Coltheart Varieties of developmental dyslexia. *Cognition* 47(2):149-180 (1993).
41. C. Dollaghan & T.F. Campbell Nonword repetition and child language impairment. *Journal of Speech, Language, and Hearing Research* 41(5):1136-1146 (1998).
42. A.S. Kaufman (1990) *Kaufman brief intelligence test: Kbit* (AGS, American Guidance Service Circle Pines, MN).
43. R. Olson, H. Forsberg, B. Wise, & J. Rack Measurement of word recognition, orthographic, and phonological skills. (1994).
44. R. Olson, B. Wise, F. Conners, J. Rack, & D. Fulker Specific deficits in component reading and language skills: Genetic and environmental influences. *Journal of learning disabilities* 22(6):339-348 (1989).
45. L.M. Dunn & F.C. Markwardt (1970) *Peabody individual achievement test* (American Guidance Service, Incorporated).
46. S. Jastak & G. Wilkinson Wide range achievement test-revised. Wilmington, de: Jastak associates. *City* (1984).
47. R.K. Wagner, J.K. Torgesen, C.A. Rashotte, & N.A. Pearson (1999) *Comprehensive test of phonological processing: Ctopp* (Pro-ed Austin, TX).
48. S.E. Gathercole, C.S. Willis, A.D. Baddeley, & H. Emslie The children's test of nonword repetition: A test of phonological working memory. *Memory* 2(2):103-127 (1994).
49. D. Wechsler Wechsler adult intelligence scale. *Archives of Clinical Neuropsychology* (1955).
50. B.T. Brus & M.J.M. Voeten (1973) *Een-minuut-test: Vorm a en b; schoolvorderingentest voor de technische leesvaardigheid, bestemd voor het tweede tot en met het zesde leerjaar van het basisonderwijs; verantwoording en handleiding* (Berkhout).
51. K. Van den Bos, H.L. Spelberg, A. Scheepstra, & J. De Vries De klepel. *Vorm A en B. Een test voor de leesvaardigheid van pseudowoorden. Verantwoording, handleiding, diagnostiek en behandeling* (1994).
52. C.M.P.l. Clercq, *et al.* Shortened nonword repetition task (nwr-s): A simple, quick, and less expensive outcome to identify children with combined specific language and reading impairment. *Journal of Speech, Language, and Hearing Research* 60(8):2241-2248 (2017).
53. J. Rispens & A. Baker Nonword repetition: The relative contributions of phonological short-term memory and phonological representations in children

with language and reading impairment. *Journal of speech, language, and hearing research : JSLHR* 55(3):683-694 (2012).
54. R.W. Woodcock, McGrew, K. S., and Mather, N. (2001) *Woodcock-johnson iii* (Riverside, Itasca, IL).
55. R.W. Woodcock (1987) *Woodcock reading mastery tests-revised* (American Guidance Service Circle Pines, MN).
56. S.C. Larsen & D.D. Hammill (1994) *Test of written spelling* (Pro-ed).
57. H.W. Catts, M.E. Fey, X. Zhang, & J.B. Tomblin Estimating the risk of future reading difficulties in kindergarten children. *Language, Speech, and Hearing Services in Schools* 32(1):38-50 (2001).
58. K. Landerl, *et al.* Predictors of developmental dyslexia in european orthographies with varying complexity. *J Child Psychol Psychiatry* 54(6):686-694 (2013).
59. K. Moll, *et al.* Cognitive mechanisms underlying reading and spelling development in five european orthographies. *Learning and Instruction* 29:65-77 (2014).
60. L. Verhoeven (1995) *Drie-minuten-toets* (Cito).
61. N. Bleichrodt, P. Drenth, J. Zaal, & W. Resing Revisie amsterdamse kinder intelligentie test. Instructie, normen, psychometrische gegevens. *Lisse: Swets en Zeitlinger* (1984).
62. R.C. Gur, *et al.* Age group and sex differences in performance on a computerized neurocognitive battery in children age 8-21. *Neuropsychology* 26(2):251-265 (2012).
63. J.C. Raven Coloured progressive matrices, sets a, a_b, b. *HK Lewis* (1962).
64. S. Paracchini, *et al.* Analysis of dyslexia candidate genes in the raine cohort representing the general australian population. *Genes, brain, and behavior* 10(2):158-165 (2011).
65. D. Wechsler (1992) Wechsler individual achievement test. (San Antonio, TX: Psychological Corporation).
